# Supplementary material for: Open-circuit and short-circuit loss management in wide-gap perovskite p-i-n solar cells
Source: Nat Commun. 2023 Feb 20;14:932. doi: 10.1038/s41467-023-36141-8 (PMC9941504; doi:10.1038/s41467-023-36141-8)
Supplement: Supplementary file 1 — Supplementary Information [file 41467_2023_36141_MOESM1_ESM.pdf]

SUPPLEMENTARY INFORMATION

Open-circuit and short-circuit loss management in wide-gap perovskite p-i-n solar cells

Pietro Caprioglio, Joel A. Smith, Robert D. J. Oliver, Akash Dasgupta, Saqlain Choudhary, Michael D. Farrar, Alexandra J. Ramadan, Yen-Hung Lin, M. Greyson Christoforo, James M. Ball, Jonas Diekmann, Jarla Thiesbrummel, Karl-Augustin Zaininger, Xinyi Shen, Michael B. Johnston, Dieter Neher, Martin Stolterfoht, Henry J. Snaith

Outline:

Supplementary Note 1: Drift-diffusion simulations
Supplementary Note 2: Additional JV data
Supplementary Note 3: Additional bandgap data
Supplementary Note 4: Additional J_{SC} decay data
Supplementary Note 5: Additional structural characterization
Supplementary Note 6: Additional PL
Supplementary Note 7: Calculation of the non-radiative losses
Supplementary Note 8: Intensity dependent TRPL
Supplementary Note 9: PLQY-QFLS maps
Supplementary Note 10: Transient drift-diffusion simulations
Supplementary Note 11: Additional degradation study

Supplementary Note 1: Drift-diffusion simulations

Table S1: *Drift-diffusion simulation parameters for the model device simulating a 1.8 eV perovskite utilizing PTAA as HTL and PCBM as ETL.*

**Parameter**	**Symbol**	**Value**	**Unit**
Majority carrier band offset between perovskite and PCBM	$\Delta E_{\mathrm{maj,c}}$	0 .15	eV
Majority carrier band offset between perovskite and PTAA	$\Delta E_{\mathrm{maj,v}}$	0 .1	eV
Lifetime in perovskite	τ_{pero}	1000	ns
Lifetime in PTAA	τ_p	1	ns
Lifetime in PCBM	τ_n	1	ns
Ionized acceptors in PTAA	$N_{\mathrm{A},p}^-$	$1\mathrm{x}10^5$	cm^{-3}
Ionized donors in PCBM	$N_{\mathrm{D},n}^+$	$1\mathrm{x}10^5$	cm^{-3}
Intrinsic carrier density in perovskite	N_{i}	$1\mathrm{x}10^{10}$	cm^{-3}
Minority carrier recombination velocity from perovskite to PTAA	S_1	1000	cm/s
Minority carrier recombination velocity from perovskite to PCBM	S_2	5000	cm/s
Thickness of HTL	d_{PTAA}	10	nm
Thickness of perovskite	d_{pero}	400	nm
Thickness of PCBM	d_{PCBM}	30	nm
Offset between metal and PTAA	$\Delta E_{\mathrm{F,metal}-p}$	0.2	eV
Offset between metal and PCBM	$\Delta E_{\mathrm{F,metal}-n}$	0.05	eV
Device built-in voltage	V_{BI}	1.3	V
Electron affinity PTAA	$E_{\mathrm{A,PTAA}}$	2.6	eV
Bandgap PTAA	$E_{\mathrm{G,PTAA}}$	3	eV
Electron affinity perovskite	$E_{\mathrm{A,pero}}$	3.9	eV
Bandgap perovskite	$E_{\mathrm{G,pero}}$	1.8	eV
Electron affinity PCBM	$E_{\mathrm{A,PCBM}}$	4.05	eV
Bandgap PCBM	$E_{\mathrm{G,PCBM}}$	2	eV
Electron mobility in PCBM	$\mu_{n,\mathrm{PTAA}}$	$1\mathrm{x}10^{-2}$	$\mathrm{cm}^2/\mathrm{Vs}$
Hole mobility in PTAA	$\mu_{p,\mathrm{PTAA}}$	$1\mathrm{x}10^{-5}$	$\mathrm{cm}^2/\mathrm{Vs}$
Hole mobility in P3HT	$\mu_{p,\mathrm{PTAA}}$	$1\mathrm{x}10^{-4}$	$\mathrm{cm}^2/\mathrm{Vs}$
Hole mobility in perovskite	$\mu_{p,\mathrm{pero}}$	10	$\mathrm{cm}^2/\mathrm{Vs}$
relative dielectric constant HTL	ϵ_{PTAA}	3.5	
relative dielectric constant perovskite	ϵ_{pero}	22	
relative dielectric constant PCBM	ϵ_{C60}	5	
Effective electron density of states in HTL	$N_{\mathrm{C/V,PTAA}}$	$1\mathrm{x}10^{20}$	cm^{-3}
Effective electron density of states in PCBM	$N_{\mathrm{C/V,C60}}$	$1\mathrm{x}10^{20}$	cm^{-3}
Effective electron density of states in perovskite	$N_{\mathrm{C/V,pero}}$	$3.1\mathrm{x}10^{18}$	cm^{-3}

All drift diffusion simulations are carried using the software SCAPS (https://scaps.elis.ugent.be/). In the simulation presented in **Fig. 2d**, the perovskite/ETL interface is modified by extending the perovskite layer with a perovskite surface featuring a slightly larger E_{G} and a lower valence band. This additional layer at the perovskite surface retains all the characteristic parameters of the perovskite but larger $E_{\mathrm{G}} = 2\ eV$.

Shockley-Read-Hall for unbalanced carrier densities

The classic Shockley-Read-Hall (SRH) recombination describes the recombination of free charges with impurity states. In the model the recombination rate is expressed with respect to the impurity density N_I, the electron and hole densities, n_e and n_h, their capture cross sections, C_e and C_h, and their de-trapping rates, β_e and β_h.

$$R = \frac{C_e C_h (n_e n_h - n_i{}^2)}{[C_e n_e + \beta_e] + [C_h n_h + \beta_h]} \cdot N_I \qquad \textit{Eq.1}$$

In the case of an intrinsic semiconductor, with negligible de-trapping rates (i.e. assuming midgap trap states), *Eq.1* can be approximated to

$$R = \frac{C_e C_h (n_e n_h)}{[C_e n_e] + [C_h n_h]} \cdot N_I \qquad \textit{Eq.2}$$

If we are now in a situation where $C_e = C_h$ but $n_e \neq n_h$ at the recombination site (e.g. across the perovskite/ETL interface), the recombination rate will be governed by the minority carriers. For example, with unbalanced carrier densities $n_h \ll n_e$, the recombination rate is governed by the minority carrier n_h and it can be rewritten as

$$R = \frac{C_e C_h (n_e n_h)}{[C_e n_e]} \cdot N_I = C_h n_h \cdot N_I \propto n_h \qquad \textit{Eq.3}$$

In the situation treated in our study, because of the high electron density present in the electron transport layer, we are assuming that all traps are filled and the rate of recombination across the interface is completely governed by the density of hole in the perovskite in the proximity of that interface.

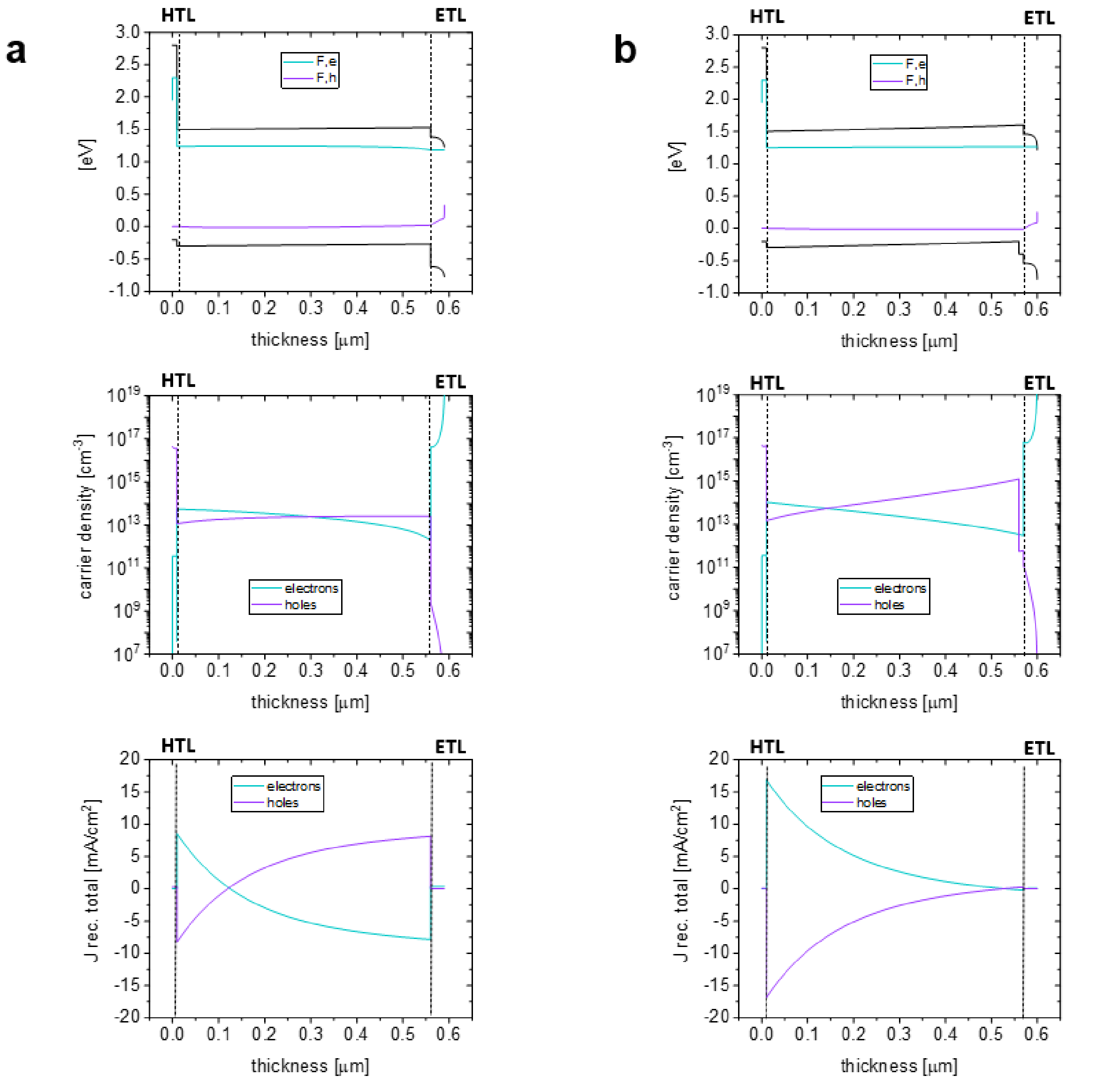

Figure S1: *Full range of drift-diffusion results presented in Fig. 1 of the main text. a) Band structure, carrier density profile and total recombination current distribution for a typical reference device. b) Same simulation results as in a) for a surface modified device.*

Supplementary Note 2: Additional JV data

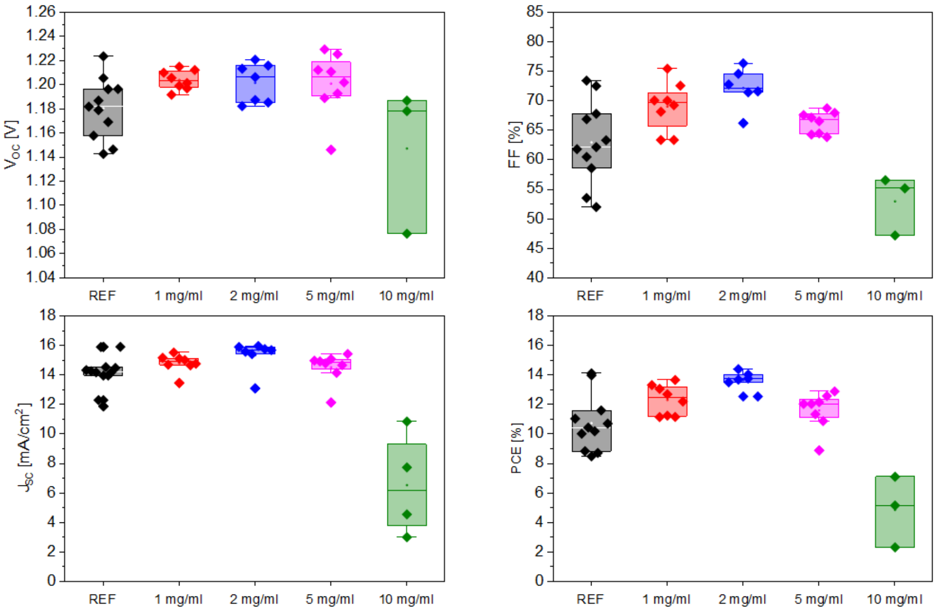

Figure S2: *Device statistics obtained from reverse JV scans with a scan rate of 0.3 V/s of a series of 1.8 eV perovskite devices using different concentrations of GuaBr as perovskite surface treatment.*

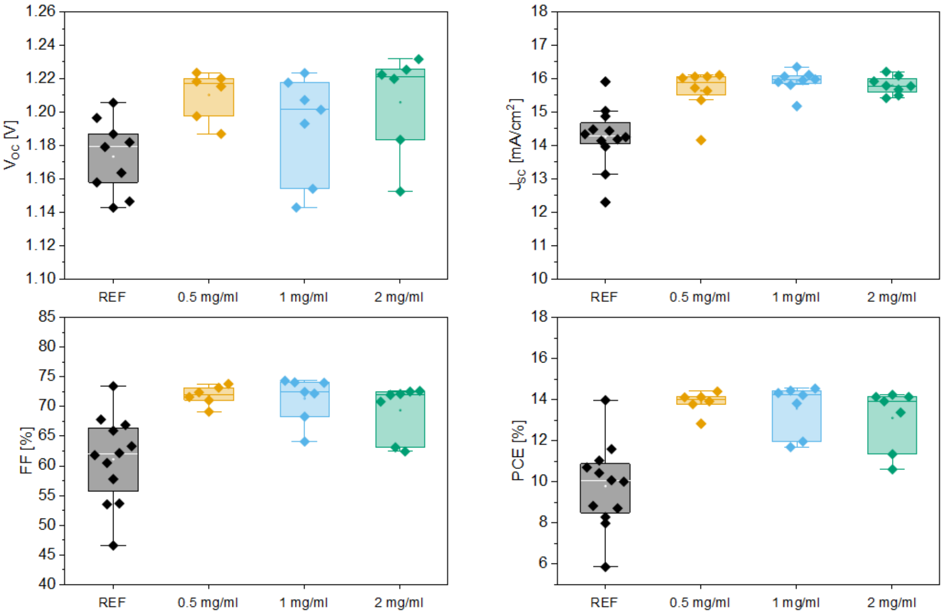

Figure S3: *Device statistics obtained from reverse JV scans with a scan rate of 0.3 V/s of a series of 1.8 eV perovskite devices using different concentrations of ImBr as perovskite surface treatment.*

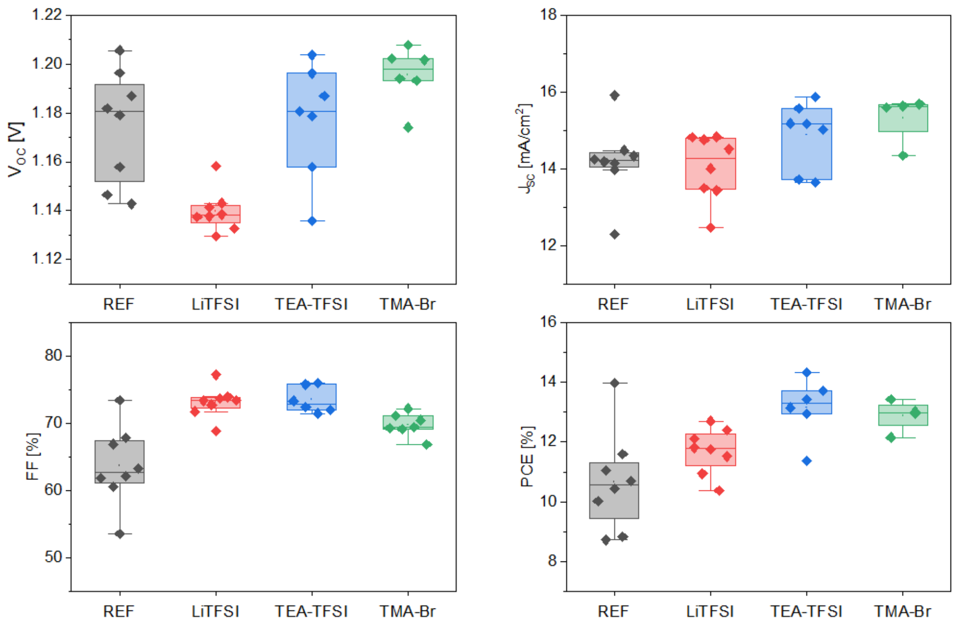

Figure S4: *Device statistics obtained from reverse JV scans with a scan rate of 0.3 V/s of a series of 1.8 eV perovskite devices using LiTFSI, TEA-TFSI or TMA-Br as PTAA surface treatment. All salts have been deposited from an IPA solution with a concentration of 2mg/ml.*

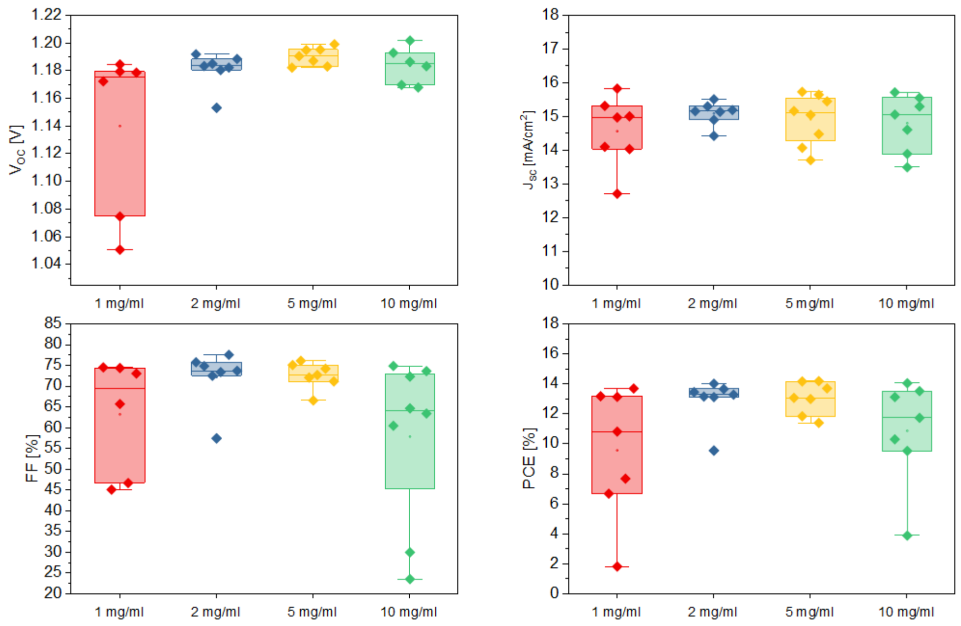

Figure S5: *Device statistics obtained from reverse JV scans with a scan rate of 0.3 V/s of initial optimization of a series of 1.8 eV perovskite devices using different concentrations of TEA-TFSI as PTAA surface treatment.*

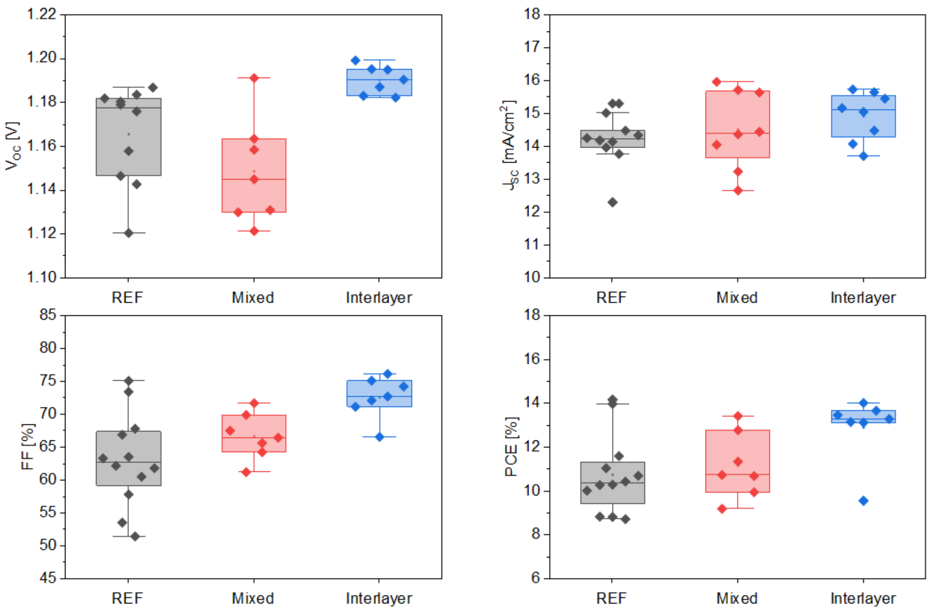

Figure S6: *Device statistics obtained from reverse JV scans with a scan rate of 0.3 V/s of a series of 1.8 eV perovskite devices testing the effect of having TEA-TFSI as PTAA surface treatment or mixed into the perovskite precursor solution.*

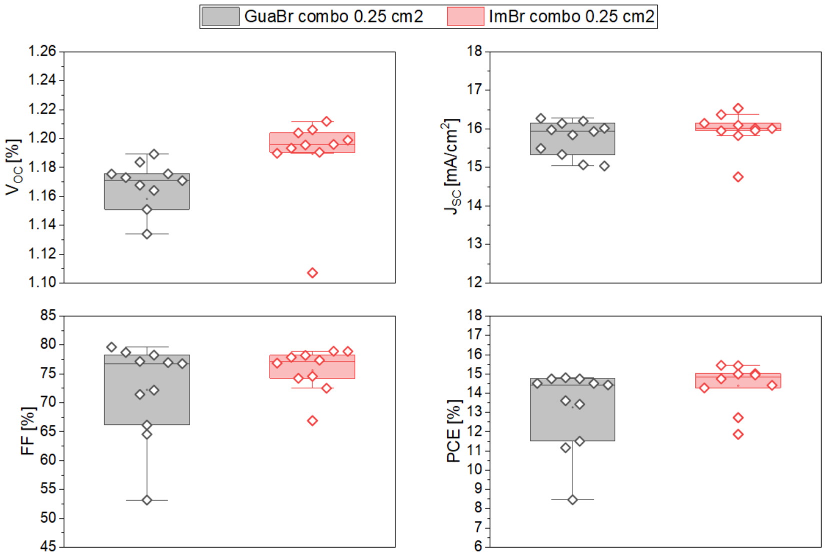

Figure S7: *Device statistics obtained from reverse JV scans with a scan rate of 0.3 V/s of a series of 1.8 eV perovskite devices using TEA-TFSI modified PTAA and either GuaBr or ImBr as perovskite surface treatment.*

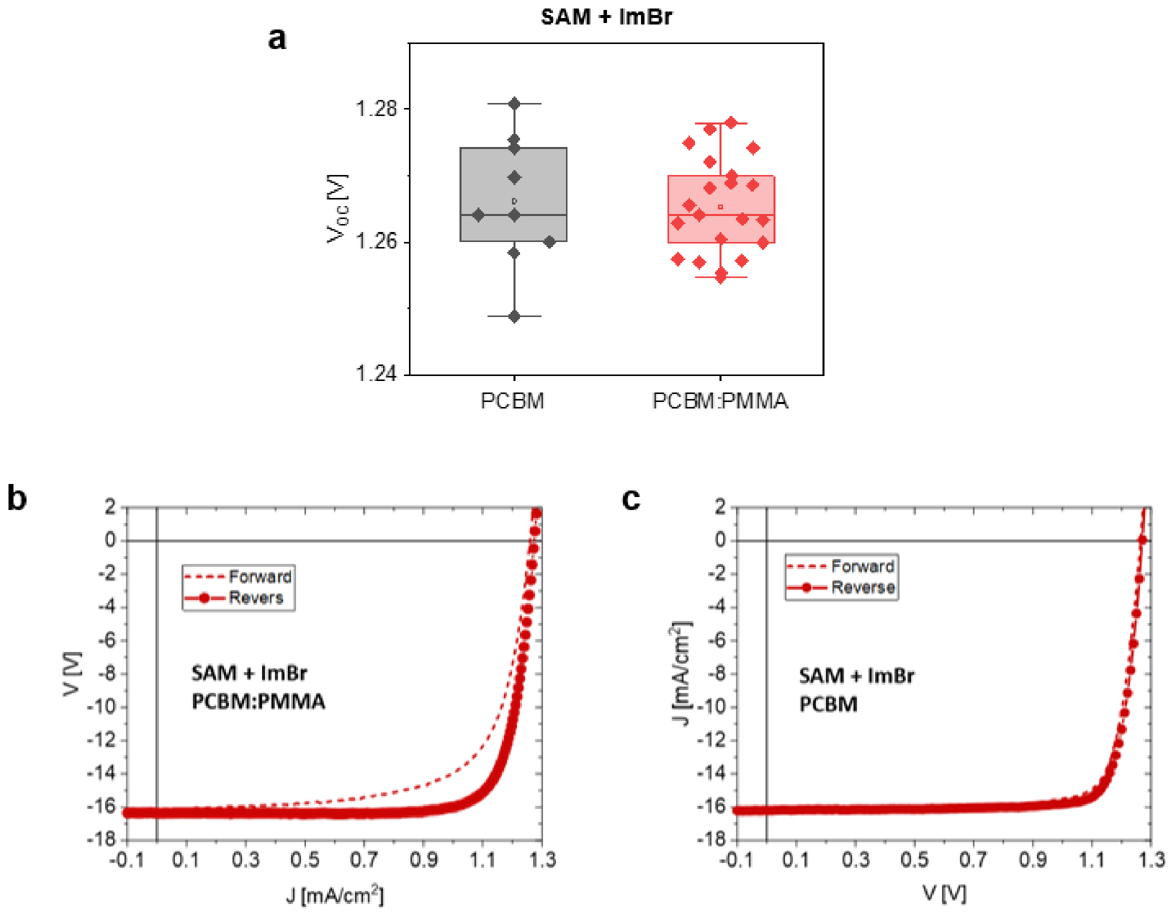

Figure S8: *a) Effect of PCBM vs. PCBM:PMMA blend on the Voc of ImBr optimized devices. The effect of the PCBM:PMMA blend is not visible anymore when the ETL interface is already fully optimized by the ImBr surface treatment. b)-c) Effect of PCBM vs. PCBM:PMMA on the hysteresis of the SAM +ImBr devices. The use of neat PCBM helps to reduce the hysteresis of the device.*

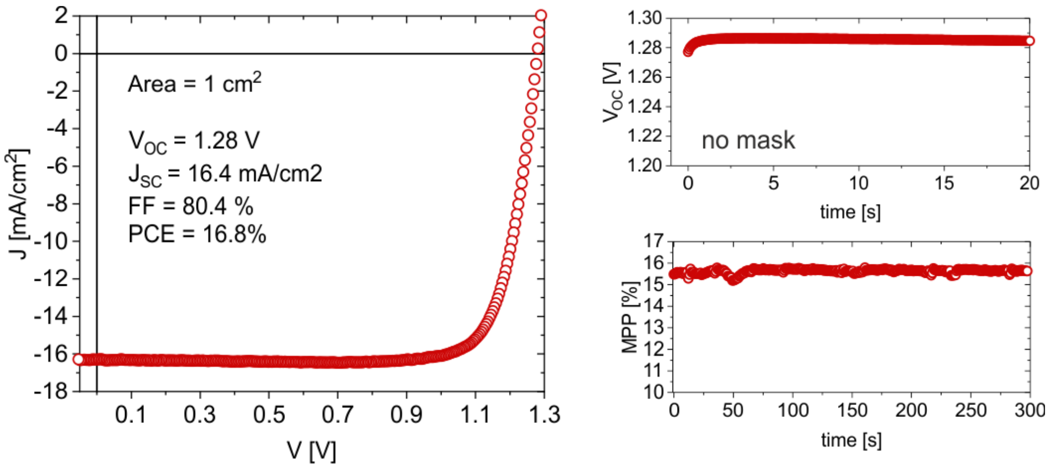

Figure S9: *Reverse JV scan with a scan rate of 0.3 V/s of the champion 1 cm^2 1.8 eV perovskite device using SAM (Me-4PACz) at the HTL and ImBr as surface treatment. The stabilized V_{OC} of the unmasked*

device is reported in order to demonstrate the maximum available device V_{OC}, which is otherwise reduced by any dark area arising from masking the pixel. The stabilized PCE under maximum power point (MPP) conditions is reported for 30 s.

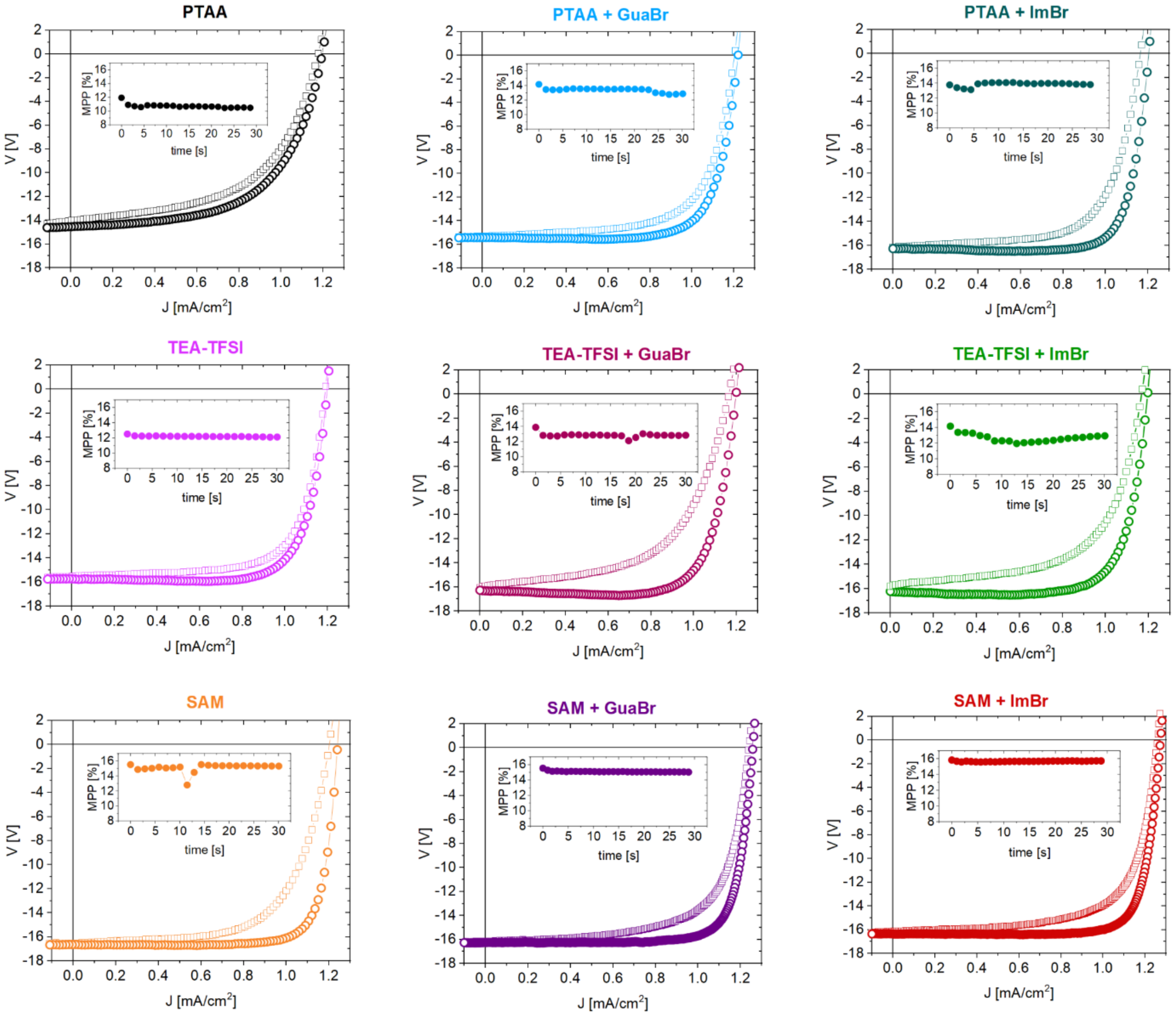

Figure S10: *Exemplary forward and reverse JV scans with a scan rate of 0.3 V/s of a series of 0.25 cm^2 1.8 eV perovskite devices representing all the different conditions investigated in this study. All devices use PCBM:PMMA as ETL. The stabilized PCE under MPP conditions is reported for each device.*

Supplementary Note 3: Additional bandgap data

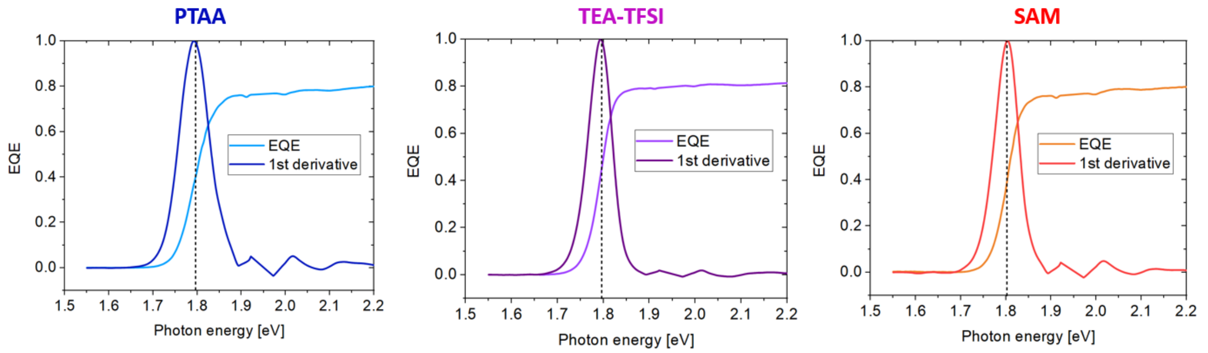

Figure S11: *Energy gap determination by looking at the inflection point of the onset of the EQE_{PV} for 1.8 eV devices using different HTLs, namely reference PTAA, TEA-TFSI treated PTAA or SAM (Me-4PACz).*

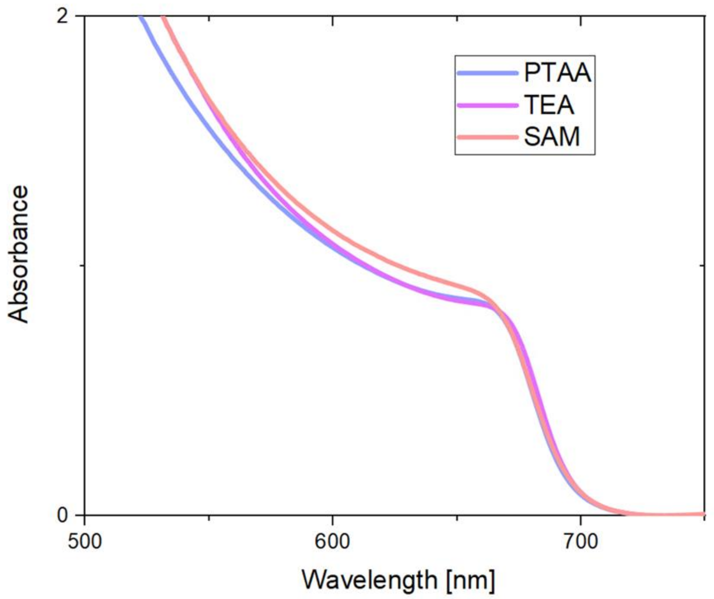

Figure S12: *Absorption spectra of perovskite films grown on top of different HTLs, namely reference PTAA, TEA-TFSI treated PTAA or SAM (Me-4PACz).*

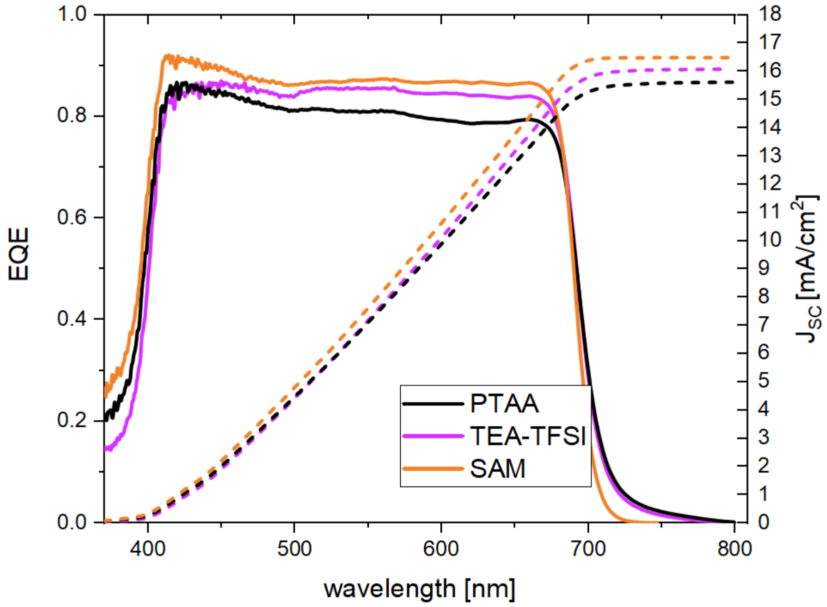

Figure S13: *EQE_{PV} and integrated J_{SC} for 1.8 eV devices using different HTLs, namely reference PTAA, TEA-TFSI treated PTAA or SAM (Me-4PACz). The integrated J_{SC}s match within 1% with the JV scan J_{SC}s.*

We note a larger absorption at low energies for the PTAA device in this particular EQE series. We note that this small effect does not affect the absorber bandgap and is only due to batch-to-batch variation, as confirmed by the EQEs in Fig. S11.

Supplementary Note 4: Additional J_{SC} decay data

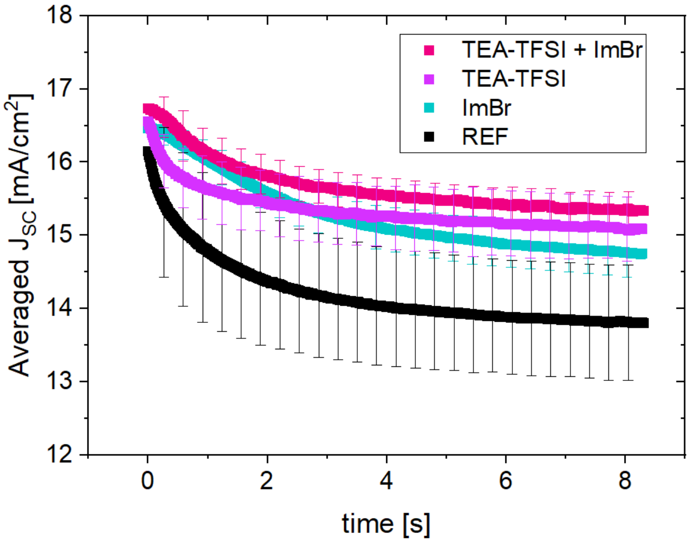

Figure S14: *J_{SC} decays over time averaged over 10-15 devices comparing our reference condition to devices using either TEA-TFSI as PTAA surface treatment, ImBr as perovskite surface treatment or the combination of both. The measurement is taken by holding the device at V_{OC} conditions and switching to J_{SC} immediately afterwards. Error bars indicate the standard deviation.*

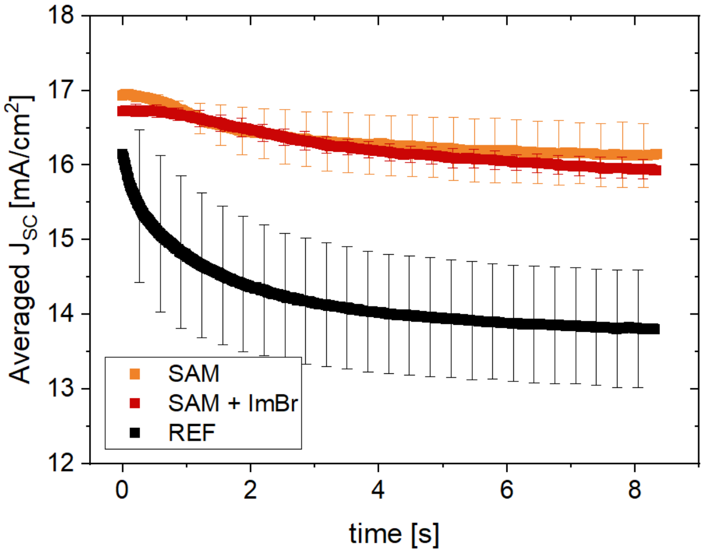

Figure S15: *J_{SC} decays over time averaged over 10-15 devices comparing our reference conditions to devices using either SAM (Me-4PACz) as HTL or the combination of SAM (Me-4PACz) and ImBr as perovskite surface treatment. The measurement is taken by holding the device at V_{OC} conditions and switching to J_{SC} immediately afterwards. Error bars indicate the standard deviation.*

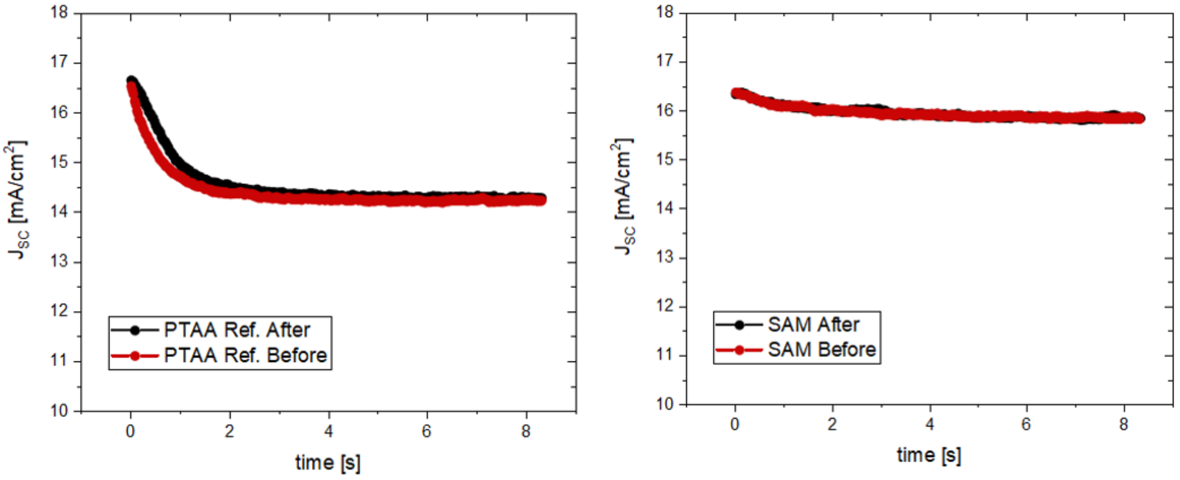

Figure S16: *J_{SC} decays for PTAA reference and SAM devices. Each device has been measured multiple times in a consequent way in order to check the reversibility of this effect. These measurements confirm that the J_{SC} decays are fully reversible and not associated to any permanent degradation process.*

Supplementary Note 5: Additional structural characterization

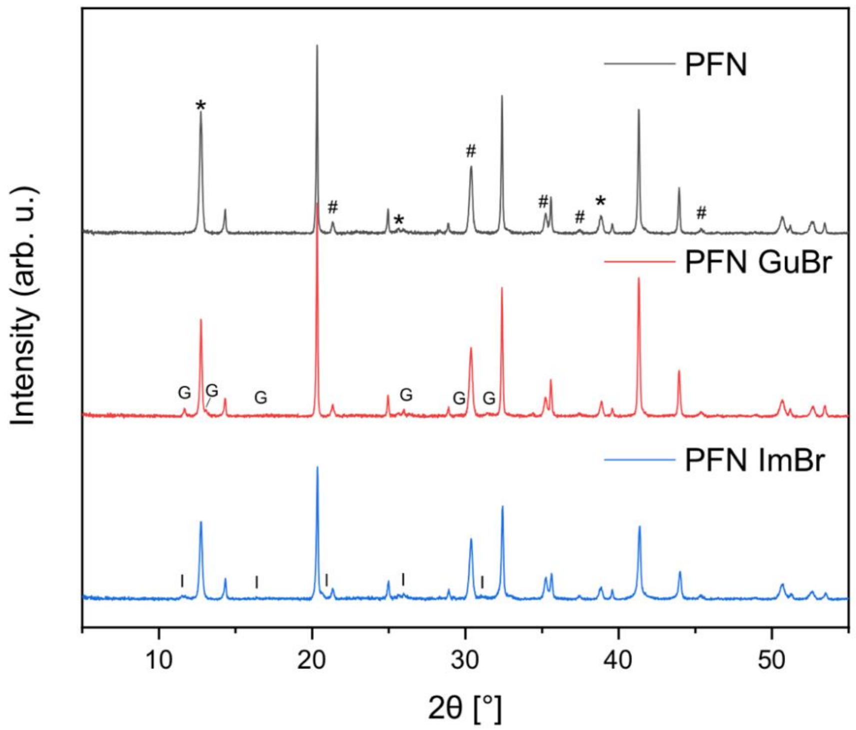

Figure S17: *XRD patterns for neat PTAA/perovskite samples as well as treated with GuaBr or ImBr. PbI_2 is marked with *, ITO with #, GuaBr-induced phase with 'G' and ImBr-induced phase with 'I'.*

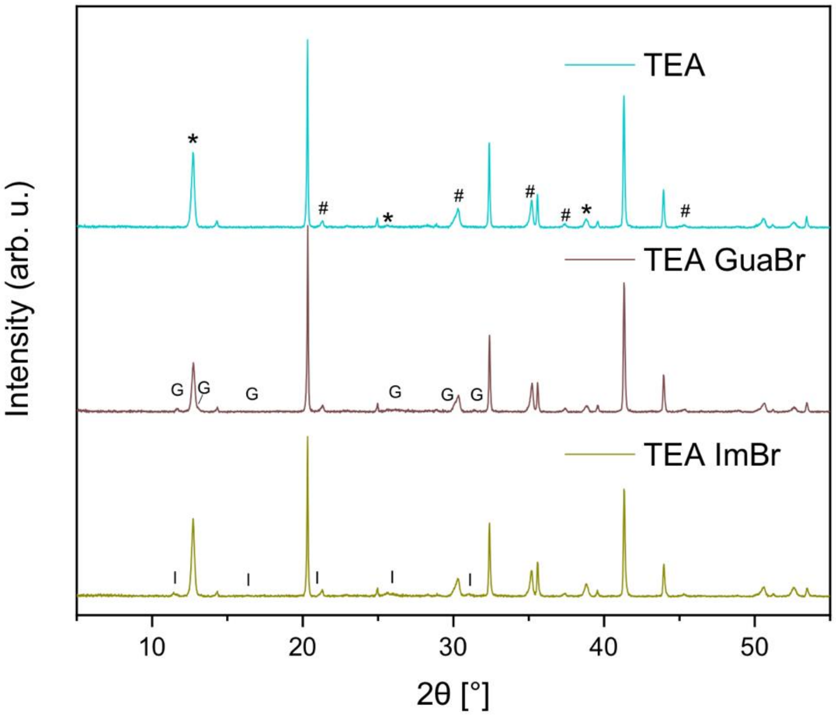

Figure S18: *XRD patterns for neat TEA-TFSI/perovskite samples and following treatment with GuaBr or ImBr.* PbI_2 *is marked with *, ITO with #, GuaBr-induced phase with 'G' and ImBr-induced phase with 'I'.*

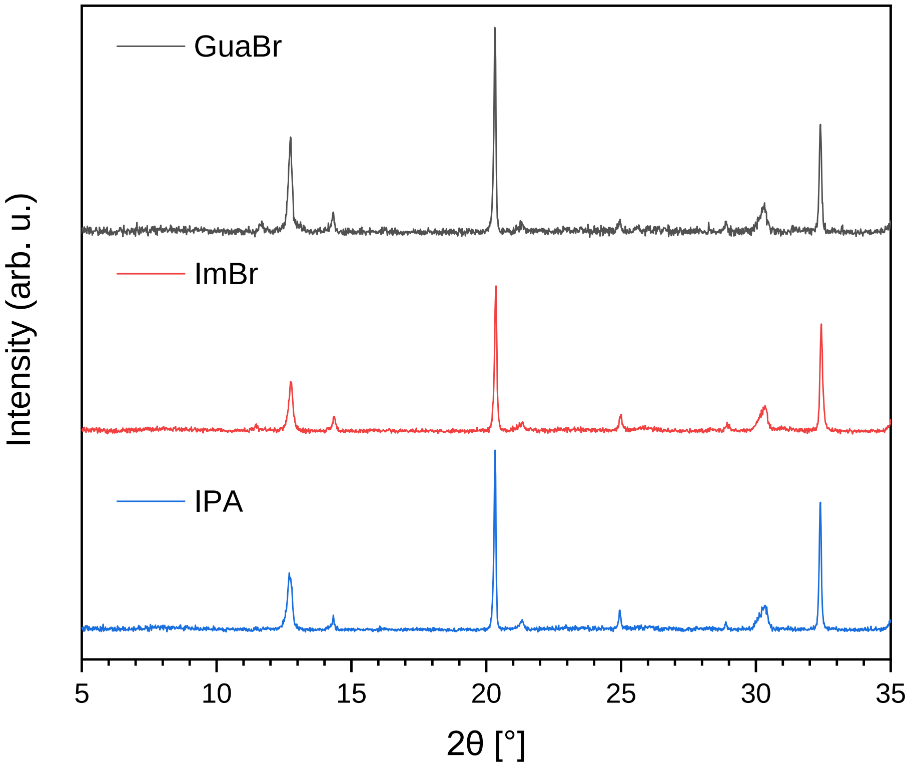

Figure S19: *XRD patterns for films in the same batch which were treated with GuaBr, ImBr or neat isopropyl alcohol, confirming no secondary phase formation with the treatment solvent.*

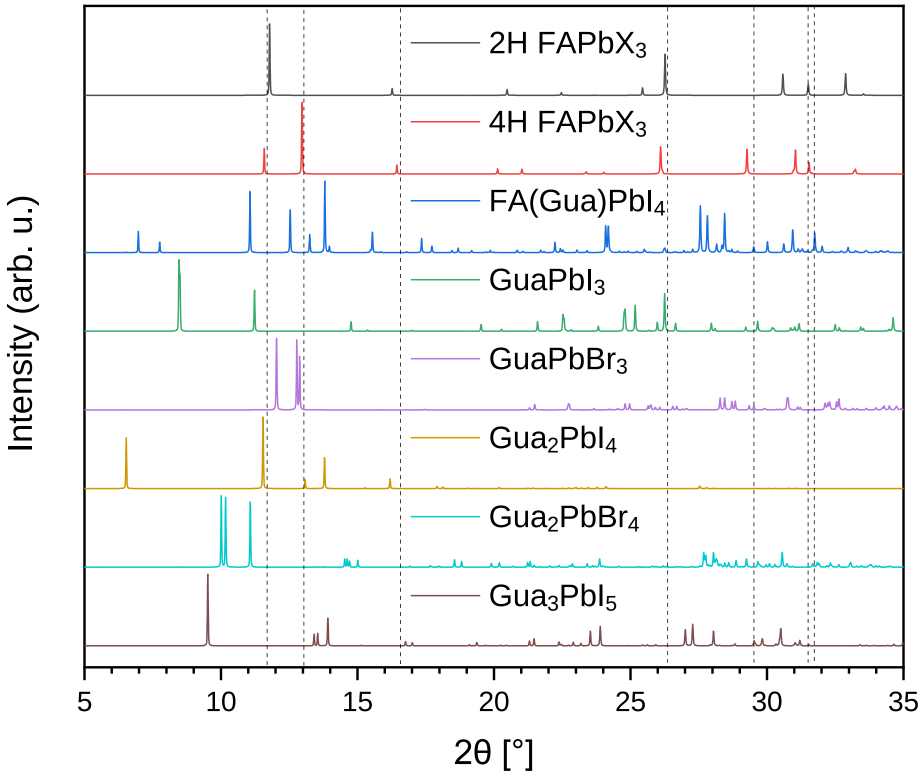

Figure S20: *Simulated XRD patterns for previously reported polytype and guanidinium containing phases. Patterns were calculated using crystallographic information in the cited works: 2H and 4H* $FAPbX_3$,[1] $FA(Gua)PbI_4$,[2] $GuaPbI_3$,[3] $GuaPbBr_3$ *(DFT calculated structure),*[4] Gua_2PbI_4 *(PDF Card - 04-013-4715),*[5] Gua_2PbBr_4,[6] *and* Gua_3PbI_5.[7] *We thank Prof. Gustavo de Miguel and Dr Sudip Chakraborty for providing the structural data for* $GuaPbI_3$ *and* $GuaPbBr_3$ *respectively.*

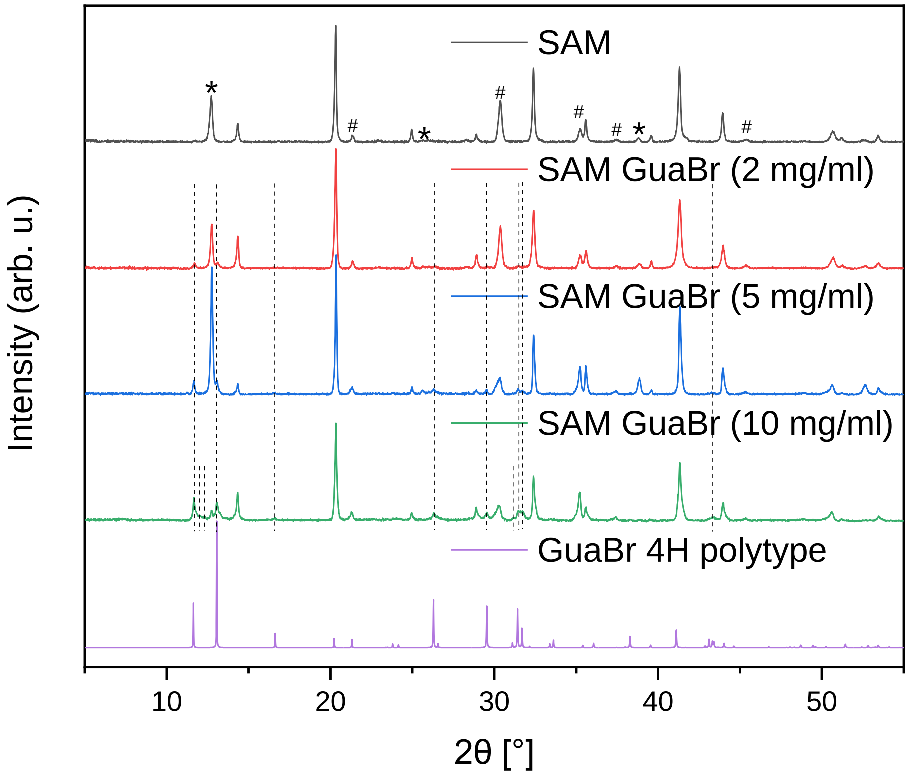

Figure S21: *XRD patterns for SAM/perovskite samples treated with a range of GuaBr concentrations (2, 5, 10 mg/ml). GuaBr-induced phase peaks are marked with dashed lines. At the highest concentration of GuaBr treatment (10 mg/ml), additional peaks emerge which could relate to a 6H polytype. Simulated diffraction pattern is shown for a GuaBr 4H polytype with lattice parameters a = b = 8.77(57) Å, c = 14.94(28) Å (β=120°), which was calculated in VESTA [8] by distorting the unit cell of a mixed-halide 4H polytype as reported by Gratia et al.*[1]

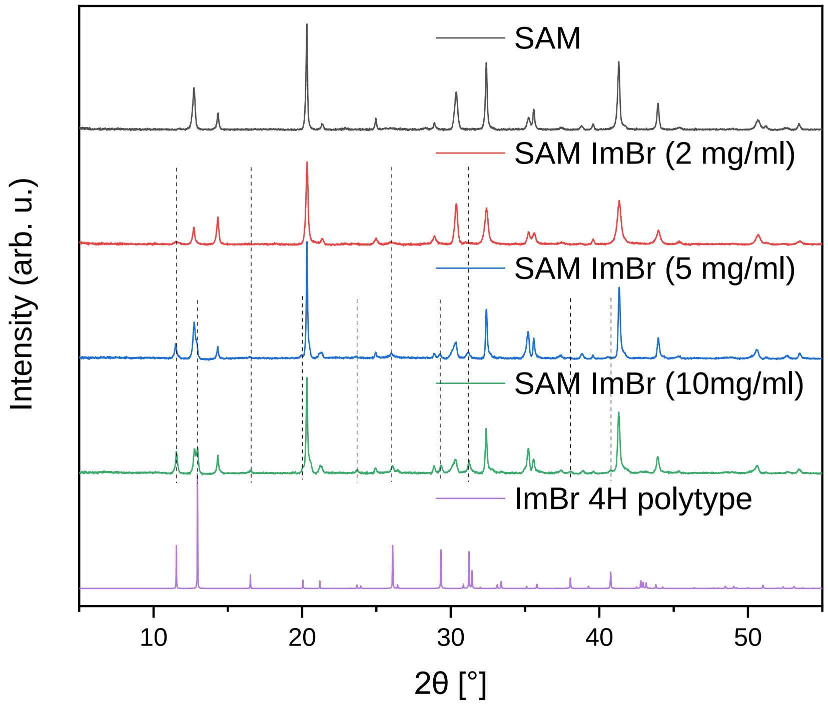

Figure S22: *XRD patterns for SAM/perovskite samples treated with ImBr with different concentrations (2, 5, 10 mg/ml). ImBr-induced phase peaks are marked with dashed lines. For the lowest ImBr concentration, some characteristic 4H peaks are absent, which could point to the phase having greater face-sharing octahedral connectivity, closer to a 2H polytype (δ-phase). Over 5 mg/ml, additional peaks related to the 4H polytype phase become clear. The simulated diffraction pattern for a ImBr-induced 4H polytype is also shown, with lattice parameters a = b = 8.84(73) Å, c = 15.01(15) Å (β = 120°). This was calculated by modifying the lattice parameters of a mixed-halide 4H polytype previously reported by Gratia et al.*[1]

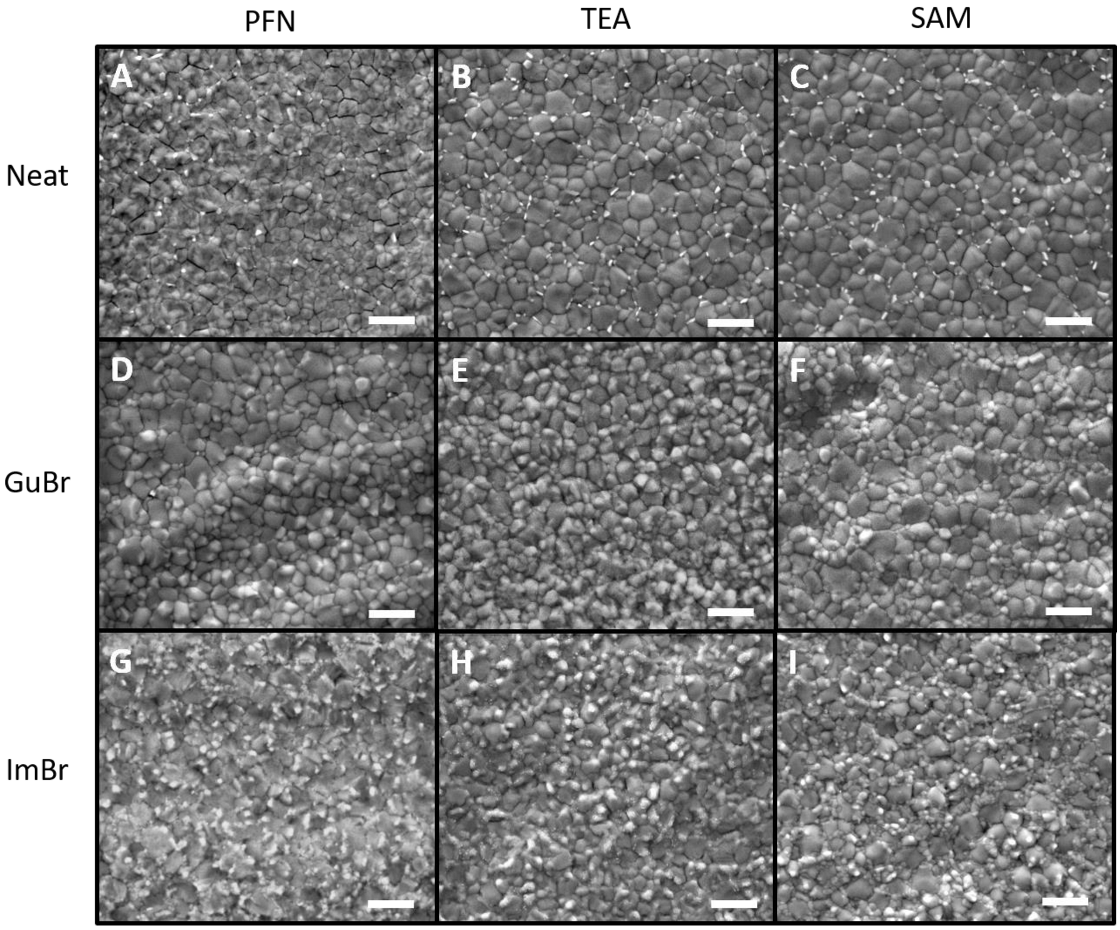

Figure S23: *Scanning Electron Microscopy images of perovskite film deposited on PTAA-PFN, TEA-TFSI and SAM. The different surface treatments with GuaBr and ImBr are shown. A-C) Unpassivated perovskite film deposited on PFN, TEA and SAM respectively. D-F) GuaBr passivated perovskite film deposited on PFN, TEA and SAM respectively. G-I) ImBr passivated perovskite film deposited on PFN, TEA and SAM respectively.*

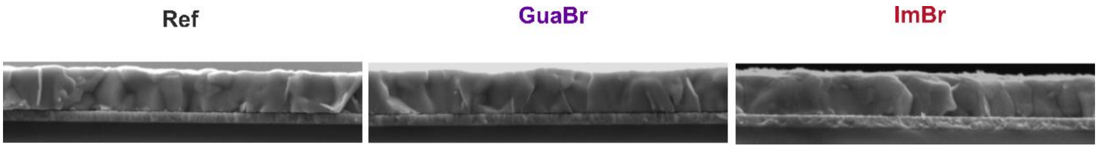

Figure S24: *Cross-sectional SEM images for ITO/SAM/perovskite samples with a) no treatment, b) GuaBr treatment and c) ImBr treatment. These immages discern negligable alteration on the resolvable lengthscale of the perovskite bulk grain structure with the treatments.*

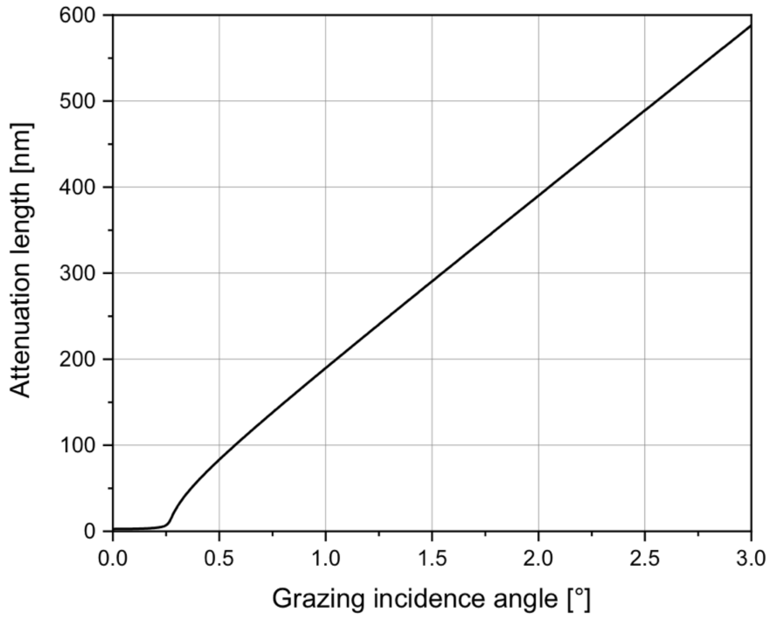

Figure S25: *Calculated X-ray attenuation length normal to a perovskite film surface as a function of the grazing angle (α_i). Calculated for CuKα radiation incident on a $FA_{0.83}Cs_{0.17}PbI_{1.8}Br_{1.2}$ with an estimated density of 4.1 gcm^{-3}.[9] For the α_i = 1°, the penetration depth (where the intensity has reduced to 1/e) is found to be 190 nm.*

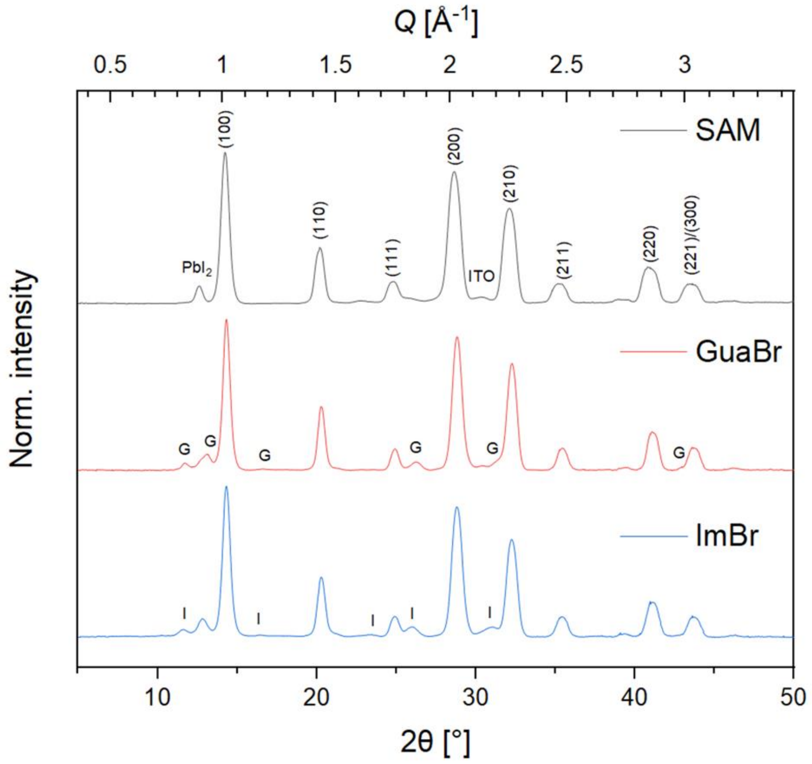

Figure S26: *Azimuthally integrated 1D GIWAXS from the 2D patterns shown in Fig 2. GuaBr-induced peaks are marked with 'G' and ImBr with 'I'. Comparing intensities of the new peaks to the perovskite (110) reflection.*

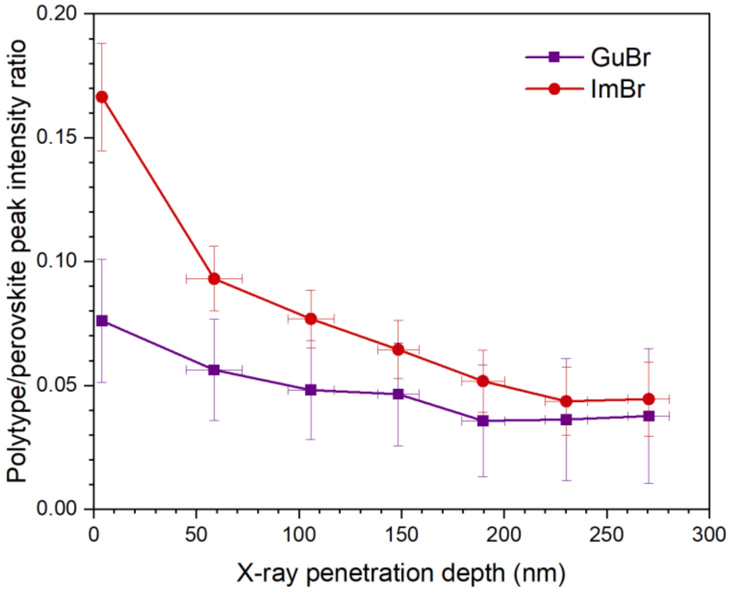

Figure S27: *Variable incidence angle GIWAXS measurements of ImBr and GuaBr-treated ITO/SAM/perovskite samples measured at* α_i *= 0.2°-1.4°. The ratio of integrated peak intensity of polytype (2θ = 10.8-12.4°) and perovskite phase (2θ = 13.5-15.5°) peaks are plotted as a function of calculated penetration depth (given in Figure S25) from 1D azimuthally integrated background-subtracted data.*

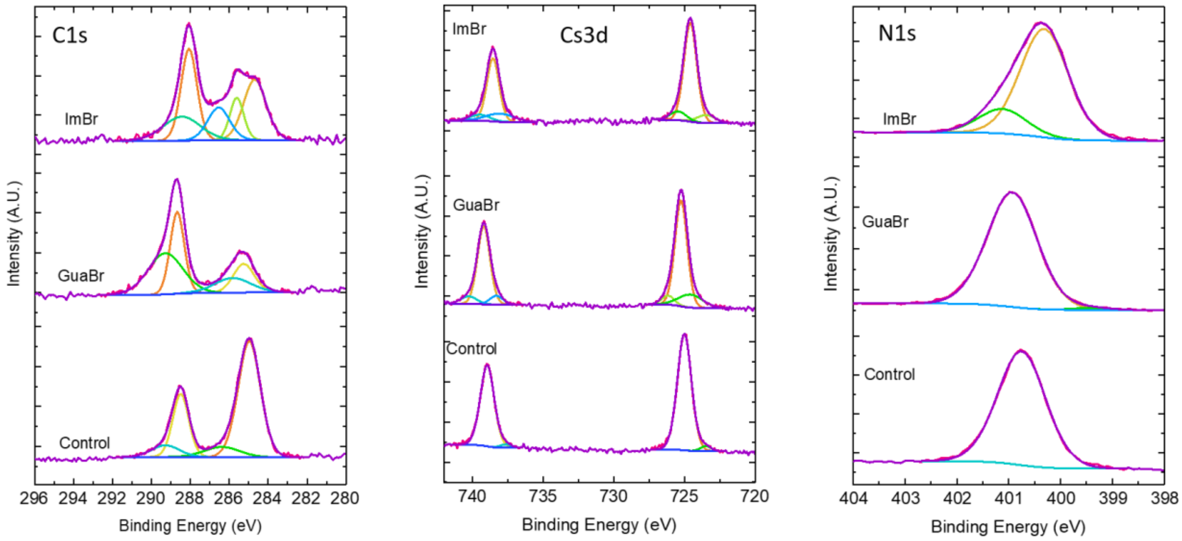

Figure S28: *High resolution X-ray Photoelectron Spectroscopy (XPS) scans for reference perovskite film, GuaBr treated or ImBr treated. Elemental regions studied are indicated within the figure.*

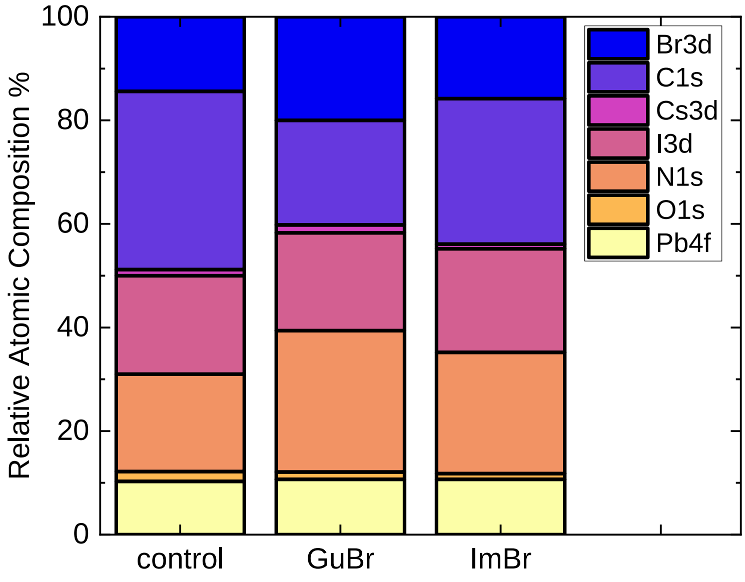

Figure S29: *XPS compositional analysis obtained from survey scans.*

Supplementary Note 6: Additional PL

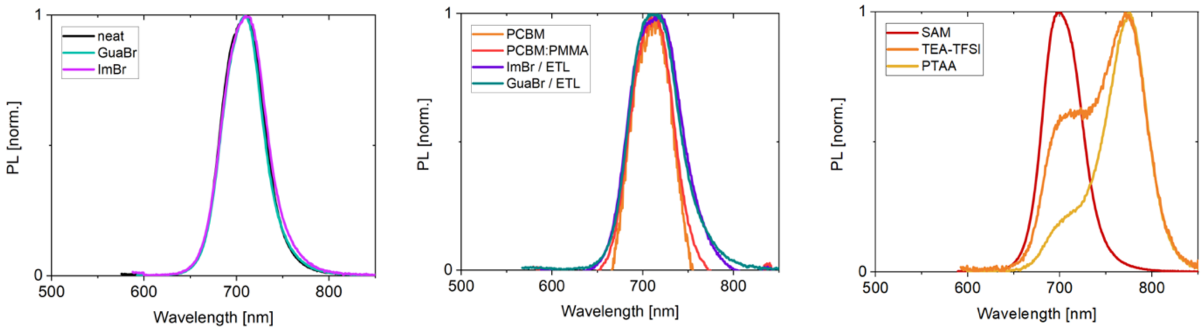

Figure S30: *Comparison of normalized PL spectra for each condition reported in Fig. 3a in order to investigate the possible presence of halide-segregation or bandgap shift upon HTL or perovskite surface treatment. The perovskite films deposited on PTAA and TEA-TFSI present a significant degree of peak shift due to halide segregation after less than 10 s illumination.*

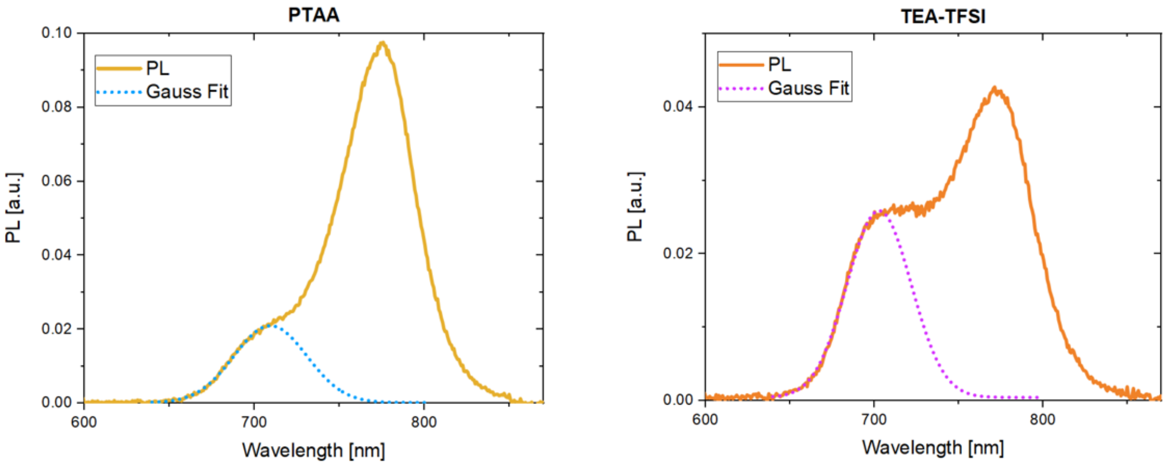

Figure S31: *Gaussian fit of the 700 nm emission for the halide-segregated sample deposited either on PTAA or TEA-TFSI. The Gaussian fit is used to estimate the component of the total PL emission that originates from radiative recombination of the mixed-phase with a bandgap of 1.8 eV bandgap. The estimated PL from the mixed-phase is used to calculate the PLQY values reported in* ***Fig. 4a****. We acknowledge that these PLQY values can possibly be affected by both non-radiative recombination and halide-segregation, therefore they represent a lower limit of the non-radiative losses. This approach follows our recent study on halide-segregation and deconvolution of different emission energies.*[10]

Supplementary Note 7: Calculation of the non-radiative losses

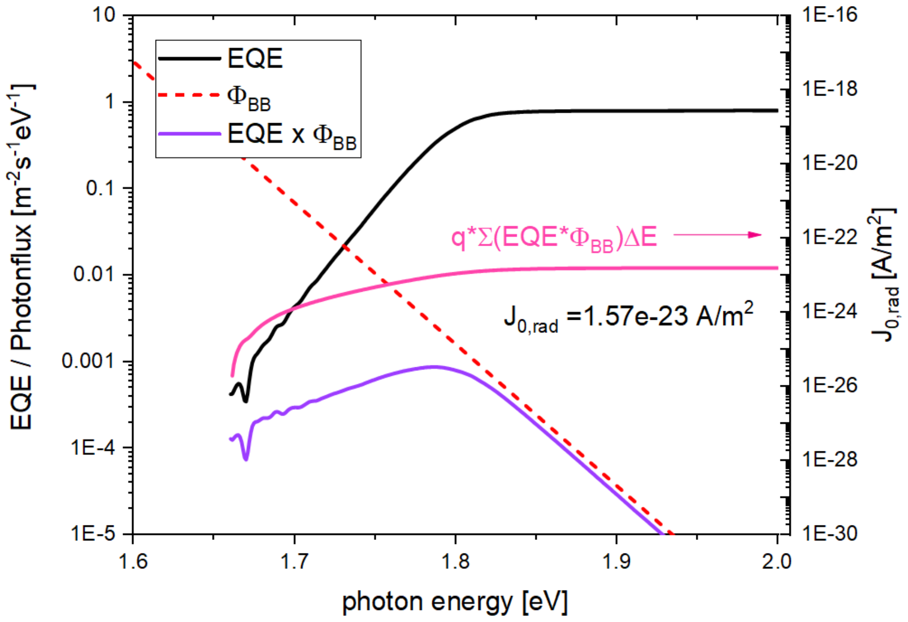

Figure S32: *EQE_{PV} onset (black line) convoluted with the black-body (ϕ_{BB}) radiation of the surroundings at 300K (red dotted line). The perovskite emission spectra resulted from the convolution is plotted in purple. The latte emission spectra is integrated over the photon energy and multiplied by the elementary charge q in order to calculate the dark radiative current $J_{0,\,rad}$, plotted in pink.*

The approach used in Fig. S32 follows the report by Rau et al. [11]. Briefly the black body photon flux is

$$\phi_{\mathrm{BB}} = \frac{1}{4\pi^2\hbar^3 c^2}\frac{E^2}{\exp\left(\frac{E}{k_{\mathrm{B}}T}\right)-1} \qquad (Eq.\ 4)$$

with ħ Plank's constant, k_B Boltzmann constant and T temperature. Assuming that the perovskite solar cell is at 300K in thermal equilibrium with its environment, the dark radiative recombination current is:

$$J_{\mathrm{em,0}} = q\int EQE_{\mathrm{PV}}(E)\phi_{\mathrm{BB}}(E)dE = J_{\mathrm{rad,0}} \qquad (Eq.\ 5)$$

with EQE_{PV} the photovoltaic *external* quantum efficiency of the perovskite solar cell and $J_{\mathrm{em,0}}$ the current giving rise to emission, which also defines the dark radiative recombination current at $V = 0$. From that, the radiative limit of the QFLS ($\mathrm{QFLS_{rad}}$) ($\mathrm{EQE_{EL}} = 1$) can be calculated with the following equation:

$$\mathrm{QFLS_{rad}} = \frac{k_{\mathrm{B}}T}{q}\ln\left(\frac{J_{\mathrm{G}}}{J_{\mathrm{rad,0}}}\cdot 1\right) \qquad (Eq.\ 6)$$

where J_{G} is the generation current under illumination, in this case approximated to the short circuit current J_{sc}.

As such we calculated the radiative V_{OC} of our perovskite absorber by using Eq. *Eq. S6*

$$V_{\mathrm{OC,rad}} = \frac{k_{\mathrm{B}}T}{q}\ln\left(\frac{J_{SC}}{J_{\mathrm{0,rad}}}\right) = 1.487\ V \qquad (Eq.\ 7)$$

Moreover, by using the PLQY measured for each different condition it is possible to calculate the induced V_{OC} with respect to the radiative V_{OC} potential

$$V_{\mathrm{OC,loss}} = V_{\mathrm{OC,rad}} + \frac{k_{\mathrm{B}}T}{q}\ln\left(\mathrm{PLQY}\right) \qquad (Eq.\ 8)$$

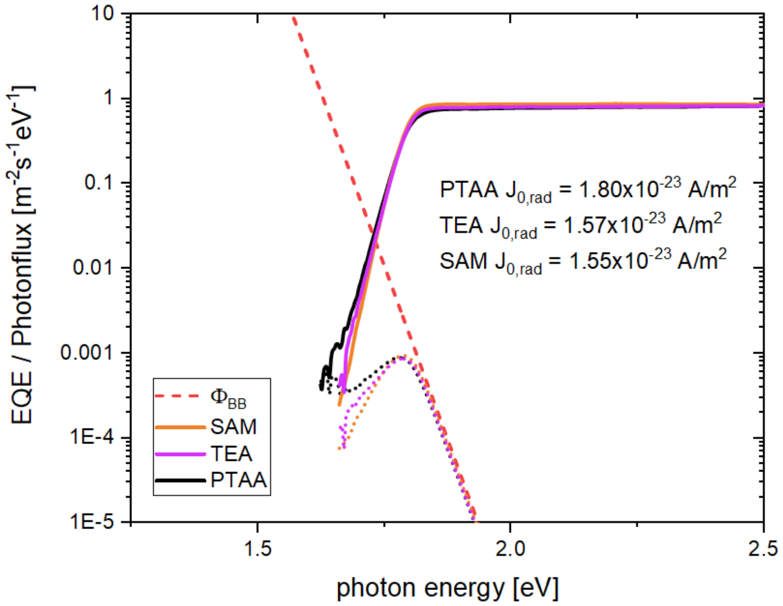

Figure S33: *$J_{0,rad}$ calculation for each individual device variation investigated. Comparing the absorption of our different devices we observe that they have almost identical contribution from tail states. As such, all devices result in very similar $J_{0,rad}$ values and the following $QFLS_{rad,\ PTAA}$ = 1.496 eV, $QFLS_{rad,\ TEA}$ = 1.499 eV and $QFLS_{rad,\ SAM}$ = 1.495 eV. Calculating individual radiative QFLS for each device shows that the variation is only in the order of 3 meV and thus negligible. Therefore, we can conclude that the influence of the tail state absorption to $J_{0,rad}$ and consequently on QFLS is almost identical for each device type investigated in our study.*

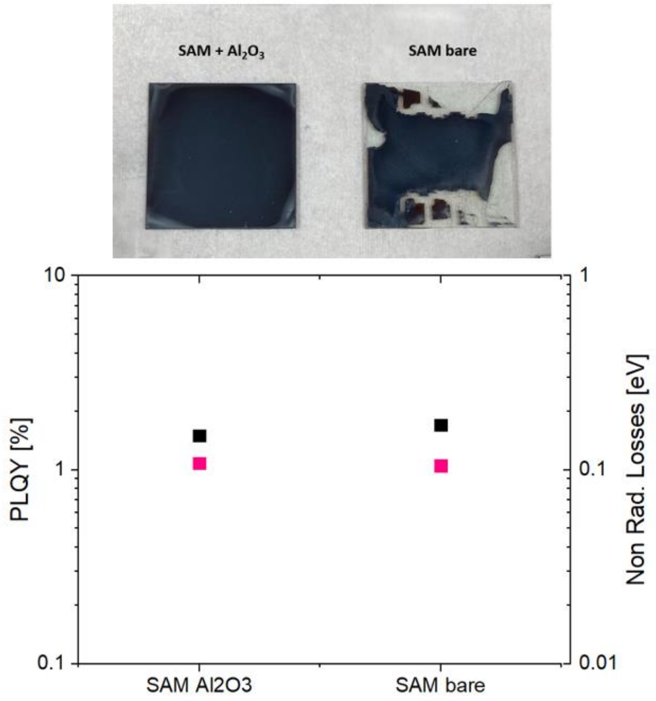

Figure S34: *On the top, picture of perovskite film spin-coated on a SAM bottom layer with or without Al_2O_3 nanoparticles treatment. On the bottom, respective PLQY and non-radiative losses calculation for the film in the picture. Both samples show very similar PLQY and non-radiative losses indicating*

no effect of the Al_2O_3 nanoparticles treatment on the recombination at this interface. The non-radiative losses have been calculated following the same approach as detailed in the next section.

Supplementary Note 8: Intensity dependent TRPL

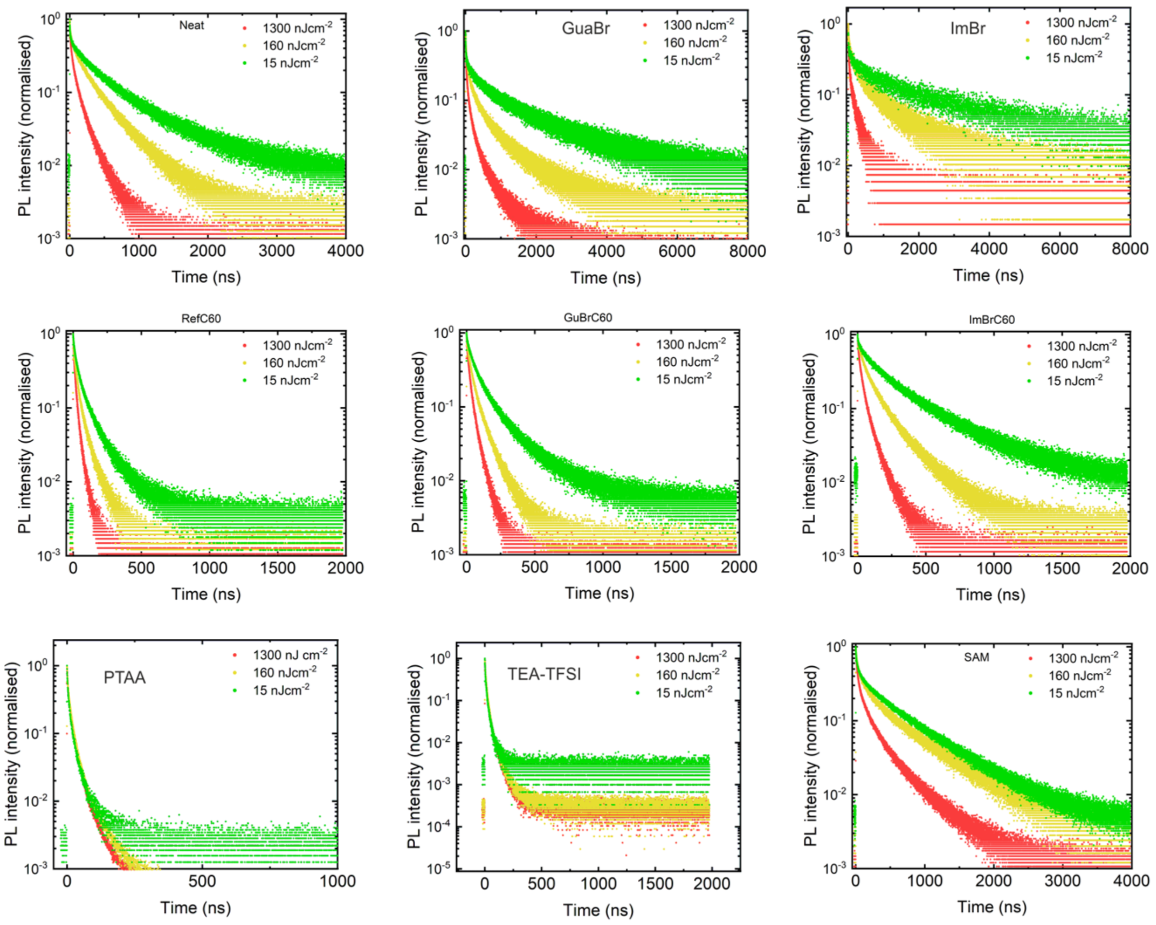

Figure S35: *Time resolved photoluminescence decays exciting at three different intensities with a 395 nm laser for all conditions investigated in this study.*

For the isolated films, a least-squares fit was carried out on the lowest-fluence PL decays using a stretched exponential of the form $I = I_0 e^{-\left(\frac{t}{\tau_{char}}\right)^{\beta}}$.[12] The average decay lifetime was then calculated as $\tau_{av} = \left(\frac{\tau_{char}}{\beta}\right)\Gamma\left(\frac{1}{\beta}\right)$, and the monomolecular recombination rate was obtained as $k_1 = \frac{1}{2\tau_{av}}$.[13]
For the films with transport layers attached, a least squares fit was carried out at each fluence using a double exponential $I = A_1 e^{-t/T1} + A_2 e^{-t/T2}$. This was necessary to account for the two separate processes that result in the PL transient, namely charge transfer into the transport layer and (interfacial) recombination. This fitting also ensures a smooth numerical derivative, which is important for the calculation of the differential lifetime. The transient PL of the half stacks is influenced by several factors. In addition to charge-carrier recombination within the perovskite itself, charge transfer to the CTL becomes important, as does interfacial recombination between the majority carrier in the CTL

with the minority carrier in the perovskite.[14,15] To disentangle these processes we define a differential lifetime which is given by

$$\tau_{\text{diff}}(t) = \left(\frac{\mathrm{d}\ln\big(\varphi(t)\big)}{\mathrm{d}t}\right)^{-1}, \tag{9}$$

where $\varphi(t)$ is the photon flux.

By combining the lifetimes obtained for the perovskite bulk and in contact with the transport layers, we calculate the lifetime associated with recombination at the perovskite/CTL heterojunctions. If we assume that the recombination at the perovskite/CTL interface is far greater than at the perovskite/quartz or perovskite/air interfaces, we can express the surface lifetime as[16–18]

$$\tau_{\text{surface}} = \frac{d}{S} + \frac{4}{D}\left(\frac{d}{\pi}\right)^2, \tag{10}$$

where d is the sample thickness, D is the diffusion coefficient and S is the surface recombination velocity. We determine D using the Einstein relation $D = \mu k_B T/q$, where k_B is Boltzmann's constant, T the absolute temperature and μ the mobility

Supplementary Note 9: PLQY-QFLS maps

All the spatially resolved data, namely QFLS maps and the extraction efficiency maps, were taken using an in house-built photoluminescence measurement setup. The setup consists of an LED illumination source, an image sensor and lens (camera) to collect and measure the photoluminescence (PL), a longpass filter to prevent stray/reflected light from the excitation source from falling onto the image sensor, and a source meter to bias the sample. This setup is able to measure spatially resolved photoluminescence from the sample, and is able to estimate PLQY under any applied bias.

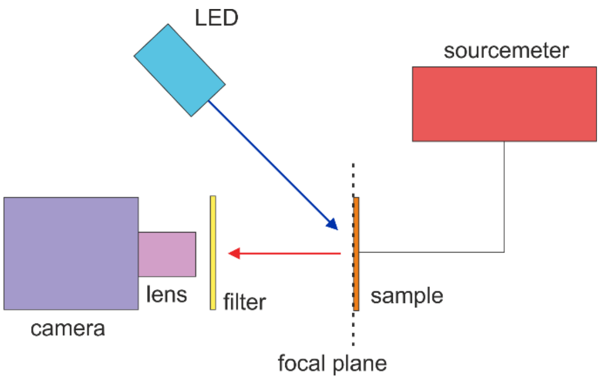

Figure S36: *Schematic representation of the PLQY imaging setup.*

The illumination was provided by a ThorLabs M450LP1 LED collimated by a Thorlabs SM2F lens. The emission was at 450 nm, well above the bandgap of the tested samples. The Intensity was controlled

by controlling the power supplied to the LED. In order to determine the ‘1 sun’ illumination, the sample was shorted and the LED power tweaked till the current readout was near the short circuit current measured on a solar simulator. The biasing was done using a Keithley.

The PL was measured using an ANDOR Zyla 4.2, a ‘scientific CMOS’ or sCMOS sensor. The sensor was cooled to 0C. The collection lens was a Kowa LM50XC, a 50mm lens with an aperture of f2.0. The sample was brought to the focal plane of the lens, and positioned to coincide with the center of the incident beam. The filter was a Thorlabs FGL515S longpass filter, with a cutoff of 515 nm. Exposure times were varied to obtain a good signal. In the analysis, the measured counts were linearly scaled by exposure time to correct for this.

In order to both correct for beam nonuniformity as well as calculating the PLQY for the QFLS calculation, a white reference was imaged without the filter. This was a barium sulphate plate, which has near unity reflectivity and a Lambertian reflection profile. This gave both a spatial map of the beam that could be used to correct the PL images, as well as the measured counts corresponding to the photon flux from the LED, at some known intensity and exposure time. The $\text{PLQY} = \frac{\text{Counts from sample}}{\text{Counts from LED}} \times C$ on a per pixel level, where C is a correction factor determined to account for:

- The LED intensity difference between the sample and the ref images
- The Quantum efficiency of the detector and wavelength response of the lens and filter.

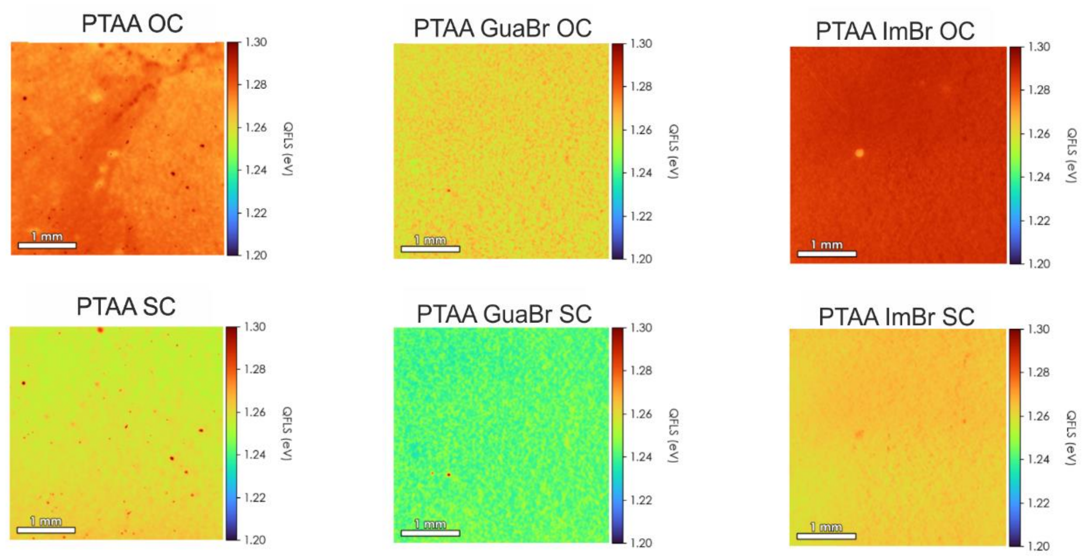

Figure S37: *Comparison of open-circuit and short-circuit QFLS maps, obtained following the method described above, of the reference PTAA device, with the same type devices either treated with GuaBr or ImBr.*

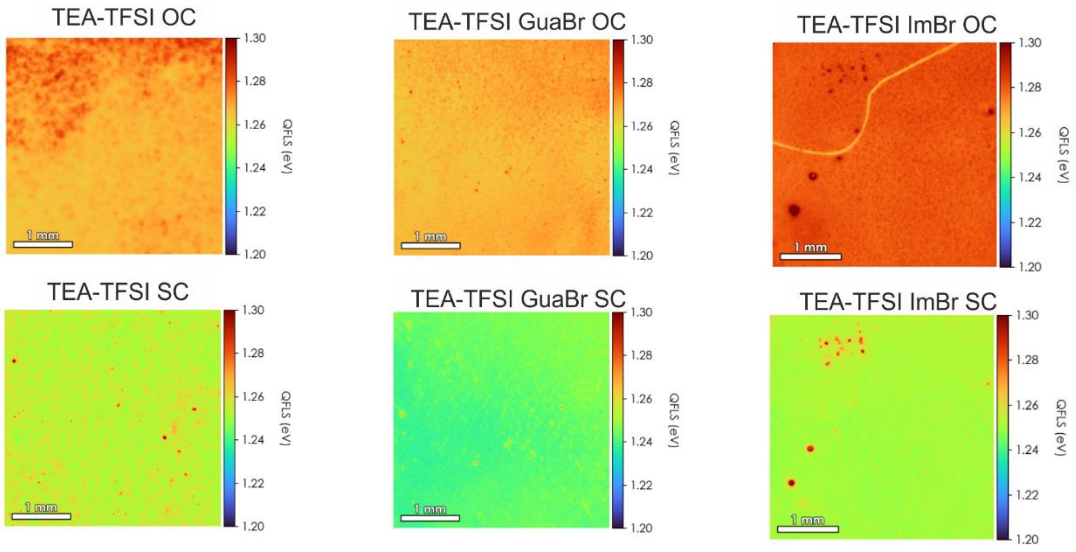

Figure S38: *Comparison of open-circuit and short-circuit QFLS maps, obtained following the method described above, of the TEA-TFSI treated PTAA device, with the same type devices either treated with GuaBr or ImBr.*

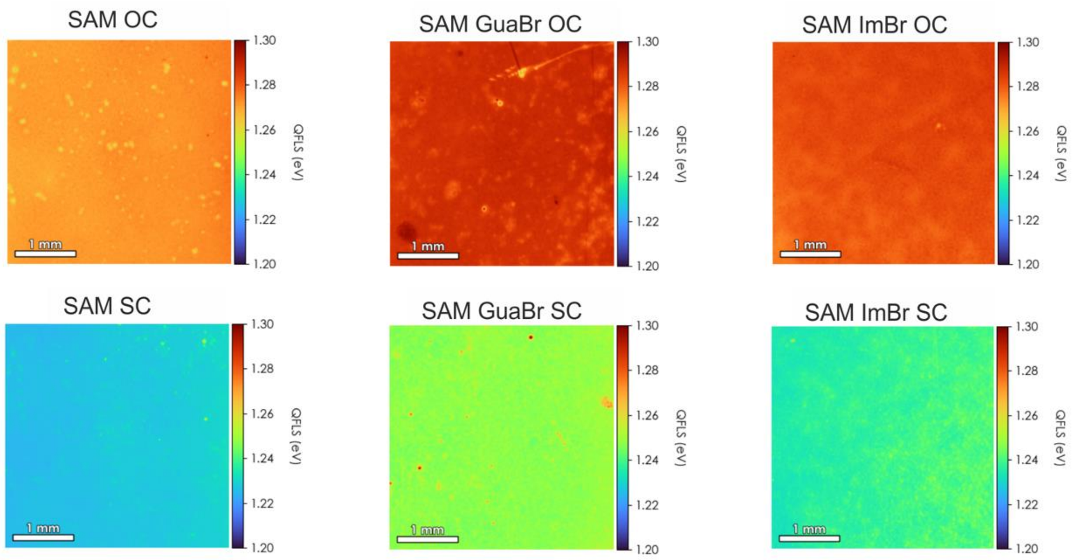

Figure S39: *Comparison of open-circuit and short-circuit QFLS maps, obtained following the method described above, of a device suing SAM (Me-4PACz) as HTL, with the same type devices either treated with GuaBr or ImBr.*

Supplementary Note 10: Transient drift-diffusion simulations

This set of transient drift-diffusion simulations was carried out using the software SETFOS developed by Fluxim.

Table S2.

Parameter	Symbol	Value	Unit	Comment
Majority carrier band offset between perovskite and PCBM	$\Delta E_{\mathrm{maj,c}}$	0	eV	
Majority carrier band offset between perovskite and PTAA	$\Delta E_{\mathrm{maj,v}}$	0.2	eV	
Lifetime in perovskite	τ_{pero}	500	ns	
Lifetime in PTAA	τ_p	1	ns	Sub-ns exciton lifetime in organic conjugated polymer, ref.[19]
Lifetime in PCBM	τ_n	1	ns	Sub-ns exciton lifetime in organic conjugated polymer, ref.[20]
Ionized acceptors in PTAA	$N_{\mathrm{A},p}^{-}$	0	cm^{-3}	
Ionized donors in PCBM	$N_{\mathrm{D},n}^{+}$	0	cm^{-3}	
Carrier recombination velocity from perovskite to the PTAA	S_{HTL}	500	cm/s	
Carrier recombination velocity from perovskite to the PCBM	S_p	1000	cm/s	
Thickness of PTAA	d_{HTL}	15	nm	
Thickness of perovskite	d_{pero}	400	nm	
Thickness of PCBM	d_{ETL}	30	nm	
Offset between metal and PTAA	$\Delta E_{\mathrm{F,metal}-p}$	0.2	eV	
Offset between metal and PCBM	$\Delta E_{\mathrm{F,metal}-n}$	0.3	eV	
Device built-in voltage	V_{BI}	1.3	V	
Bandgap PTAA	$E_{\mathrm{G,HTL}}$	3.0	eV	
Electron affinity PTAA	$E_{\mathrm{A,HTL}}$	2.5	eV	
Bandgap perovskite	$E_{\mathrm{G,pero}}$	1.8	eV	
Electron affinity perovskite	$E_{\mathrm{A,pero}}$	3.9	eV	Using photon yield spectroscopy measurements, ref.[21]
Electron affinity PCBM	$E_{\mathrm{A,ETL}}$	3.9	eV	Consider aligned C_{60} LUMO with perovskite conduction band
Bandgap PCBM	$E_{\mathrm{G,ETL}}$	2.0	eV	
Electron mobility in PCBM	$\mu_{n,\mathrm{ETL}}$	$1\mathrm{x}10^{-2}$	cm^2/Vs	$1.5\cdot10^{-2}$ cm^2/Vs measured with SCLC in ref.[22] Ref.[23] showed that $\mu_{n,\mathrm{C60}}$ and $\mu_{n,\mathrm{pero}}$ are much

				larger than $\mu_{p,\mathrm{PTAA}}$ in these devices.
Hole mobility in PTAA	$\mu_{p,\mathrm{HTL}}$	1.5×10^{-4}	cm^2/Vs	A mobility of $7.5\cdot10^{-5}$ cm^2/Vs measured on undoped PTAA with SCLC in ref.[24]
Electron mobility in perovskite	$\mu_{n,\mathrm{pero}}$	1	cm^2/Vs	
Hole mobility in perovskite	$\mu_{p,\mathrm{pero}}$	1	cm^2/Vs	
Relative dielectric constant PTAA	ϵ_{HTL}	3.5		Typical value for organic conjugated polymers.[27]
Relative dielectric constant perovskite	ϵ_{pero}	22		Typical value, e.g. ϵ_{pero} = 25 in ref.[28]
relative dielectric constant PCBM	ϵ_{ETL}	5.0		a relative permittivity of ~5 was measured at lowest frequencies (1kHz) at 300 K, ref.[29]
Effective electron density of states in PTAA	$N_{\mathrm{C/V,HTL}}$	1×10^{20}	cm^{-3}	Ref.[30]
Effective electron density of states in PCBM	$N_{\mathrm{C/V,ETL}}$	1×10^{20}	cm^{-3}	Ref.[31]
Effective electron density of states in perovskite	$N_{\mathrm{C/V,pero}}$	2.2×10^{18}	cm^{-3}	Ref.[32]
Series resistance	R_{Series}	0	Ωcm^{-2}	Previously estimated an upper limit of the ohmic series resistance to be 0.34 Ωcm^{-2} in the cell, which has a small impact on the FF.[33]
Ion density	n_{ion}	2×10^{16}	cm^{-3}	
Ion mobility	n_{ion}	10^{-8}	cm^2/Vs	
Thickness of SAM	d_{SAM}	1	nm	
Carrier recombination velocity from perovskite to the SAM	S_{SAM}	5	cm/s	
Bandgap of SAM	$E_{\mathrm{G,SAM}}$	3.0	eV	
Majority carrier band offset between perovskite and SAM	$\Delta E_{\mathrm{maj,SAM}}$	0.1	eV	
Thickness ionic interlayer	d_{TEA}	1	nm	
Ion density ionic interlayer	$n_{ion\text{-}TEA}$	2×10^{18}	cm^{-3}	

In the simulation model, we are considering the cations to be mobile, whereas the counter anions are considered to be stationary and homogenously distributed. Importantly, in the model, all layers are set to be permeable to ions.

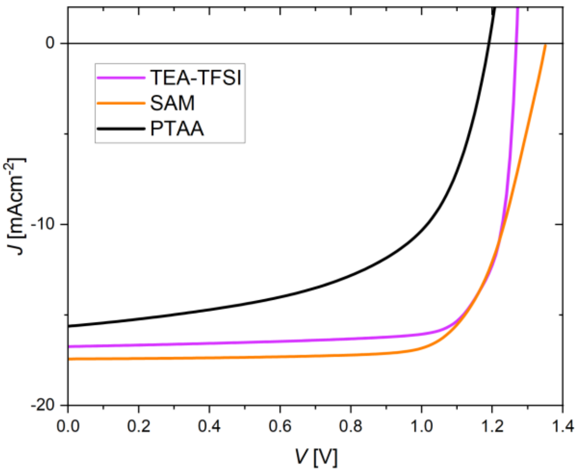

Figure S40: *Simulated JV curves modelling PTAA, TEA-TFSI and SAM cells.*

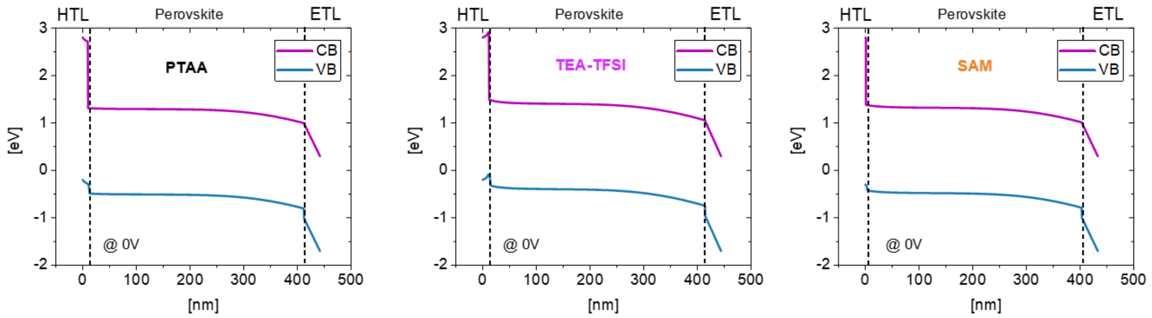

Figure. S41: *Simulated band structure at 0V conditions corresponding to the modelled devices presented in Fig. S40.*

Supplementary Note 11: Additional degradation study

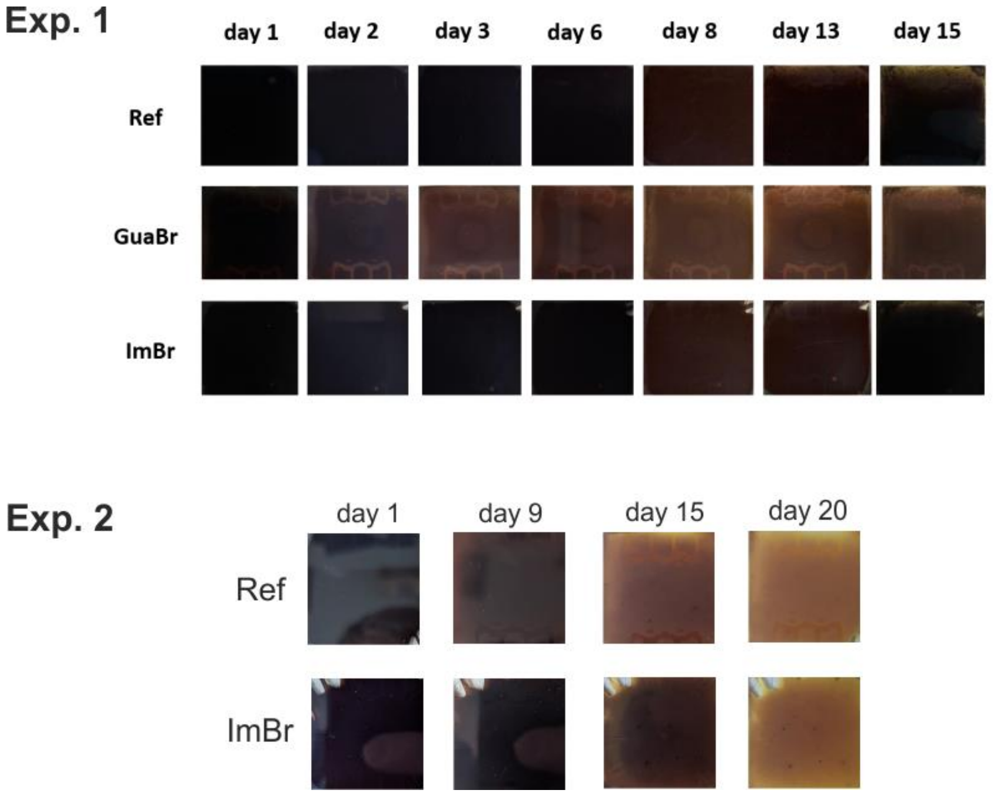

Figure S42: *Photographs of two sets of experiments ageing unencapsulated perovskite films deposited on SAM, with and without surface modifications over a period of 15-20 days. The samples were aged in air at 25 °C with a relative humidity of 40-45% under ambient indoor light.*

Supplementary References

1. Gratia, P. *et al.* The Many Faces of Mixed Ion Perovskites: Unraveling and Understanding the Crystallization Process. *ACS Energy Letters* 2686–2693 (2017) doi:10.1021/acsenergylett.7b00981.
2. Nazarenko, O. *et al.* Guanidinium-Formamidinium Lead Iodide: A Layered Perovskite-Related Compound with Red Luminescence at Room Temperature. *Journal of the American Chemical Society* **140**, 3850–3853 (2018).
3. Jodlowski, A. D., Yépez, A., Luque, R., Camacho, L. & de Miguel, G. Benign-by-Design Solventless Mechanochemical Synthesis of Three-, Two-, and One-Dimensional Hybrid Perovskites. *Angewandte Chemie - International Edition* **55**, 14972–14977 (2016).
4. Banerjee, A., Chakraborty, S. & Ahuja, R. Bromination-induced stability enhancement with a multivalley optical response signature in guanidinium [C(NH2)3]+-based hybrid perovskite solar cells. *Journal of Materials Chemistry A* **5**, 18561–18568 (2017).
5. Szafrański, M. & Katrusiak, A. Phase transitions in the layered structure of diguanidinium tetraiodoplumbate. *Physical Review B* **61**, 1026–1035 (2000).
6. Nazarenko, O. *et al.* Luminescent and Photoconductive Layered Lead Halide Perovskite Compounds Comprising Mixtures of Cesium and Guanidinium Cations. *Inorganic Chemistry* **56**, 11552–11564 (2017).
7. Deng, Z., Kieslich, G., Bristowe, P. D., Cheetham, A. K. & Sun, S. Octahedral connectivity and its role in determining the phase stabilities and electronic structures of low-dimensional, perovskite-related iodoplumbates. *APL Materials* **6**, (2018).
8. Momma, K. & Izumi, F. VESTA 3 for three-dimensional visualization of crystal, volumetric and morphology data. *urn:issn:0021-8898* **44**, 1272–1276 (2011).
9. Henke, B. L., Gullikson, E. M. & Davis, J. C. X-Ray Interactions: Photoabsorption, Scattering, Transmission, and Reflection at E = 50-30,000 eV, Z = 1-92. *Atomic Data and Nuclear Data Tables* **54**, 181–342 (1993).
10. Caprioglio, P. *et al.* Nano-emitting Heterostructures Violate Optical Reciprocity and Enable Efficient Photoluminescence in Halide-Segregated Methylammonium-Free Wide Bandgap Perovskites. *ACS Energy Letters* **6**, 419–428 (2021).
11. Rau, U. Reciprocity relation between photovoltaic quantum efficiency and electroluminescent emission of solar cells. *Phys. Rev. B* **76**, 085303 (2007).
12. Johnston, D. C. Stretched exponential relaxation arising from a continuous sum of exponential decays. *Physical Review B* **74**, 184430 (2006).
13. Richter, J. M. *et al.* Enhancing photoluminescence yields in lead halide perovskites by photon recycling and light out-coupling. *Nature Communications* **7**, 13941 (2016).
14. Kirchartz, T., Márquez, J. A., Stolterfoht, M. & Unold, T. Photoluminescence-Based Characterization of Halide Perovskites for Photovoltaics. *Advanced Energy Materials* **10**, 1904134 (2020).
15. Krogmeier, B., Staub, F., Grabowski, D., Rau, U. & Kirchartz, T. Quantitative analysis of the transient photoluminescence of CH 3 NH 3 PbI 3 /PC 61 BM heterojunctions by numerical simulations. *Sustainable Energy & Fuels* **2**, 1027–1034 (2018).
16. Sproul, A. B. Dimensionless solution of the equation describing the effect of surface recombination on carrier decay in semiconductors. *Journal of Applied Physics* **76**, 2851–2854 (1994).
17. Wang, J. *et al.* Reducing Surface Recombination Velocities at the Electrical Contacts Will Improve Perovskite Photovoltaics. *ACS Energy Letters* **4**, 222–227 (2019).

18. Stolterfoht, M. *et al.* Visualization and suppression of interfacial recombination for high-efficiency large-area pin perovskite solar cells. *Nature Energy* **3**, 847–854 (2018).
19. Dimitrov, S. D. *et al.* Singlet exciton lifetimes in conjugated polymer films for organic solar cells. *Polymers* **8**, (2016).
20. Qin, D., Gu, P., Dhar, R. S., Razavipour, S. G. & Ban, D. Measuring the exciton diffusion length of C 60 in organic planar heterojunction solar cells. *Physica Status Solidi (A) Applications and Materials Science* **208**, 1967–1971 (2011).
21. Stolterfoht, M. *et al.* The impact of energy alignment and interfacial recombination on the internal and external open-circuit voltage of perovskite solar cells. *Energy & Environmental Science* **12**, 2778–2788 (2019).
22. Mendil, N., Daoudi, M., Berkai, Z. & Belghachi, A. Disorder effect on carrier mobility in Fullerene organic semiconductor. *Journal of Physics: Conference Series* **647**, 012057 (2015).
23. Stolterfoht, M. *et al.* Approaching the fill factor Shockley–Queisser limit in stable, dopant-free triple cation perovskite solar cells. *Energy & Environmental Science* **10**, 1530–1539 (2017).
24. Luo, J. *et al.* Toward high-efficiency, hysteresis-less, stable perovskite solar cells: Unusual doping of a hole-transporting material using a fluorine-containing hydrophobic Lewis acid. *Energy and Environmental Science* **11**, 2035–2045 (2018).
25. Al-Ashouri, A. *et al.* Monolithic perovskite/silicon tandem solar cell with over 29% efficiency by enhanced hole extraction. *Science* **370**, 1300–1309 (2020).
26. Herz, L. M. Charge-Carrier Mobilities in Metal Halide Perovskites: Fundamental Mechanisms and Limits. *ACS Energy Letters* **2**, 1539–1548 (2017).
27. Torabi, S. *et al.* Strategy for enhancing the dielectric constant of organic semiconductors without sacrificing charge carrier mobility and solubility. *Advanced Functional Materials* **25**, 150–157 (2015).
28. Brivio, F., Butler, K. T., Walsh, A. & Van Schilfgaarde, M. Relativistic quasiparticle self-consistent electronic structure of hybrid halide perovskite photovoltaic absorbers. *Physical Review B - Condensed Matter and Materials Physics* **89**, 1–6 (2014).
29. Chern, G., Mathias, H., Testardi, L. R., Seger, L. & Schlenoff, J. Low-frequency dielectric permittivity of C60. *Journal of Superconductivity* **8**, 207–210 (1995).
30. Kirchartz, T. *et al.* Sensitivity of the Mott–Schottky analysis in organic solar cells. *The Journal of Physical Chemistry C* **116**, 7672–7680 (2012).
31. Kirchartz, T. *et al.* Sensitivity of the Mott–Schottky analysis in organic solar cells. *The Journal of Physical Chemistry C* **116**, 7672–7680 (2012).
32. Staub, F. *et al.* Beyond Bulk Lifetimes: Insights into Lead Halide Perovskite Films from Time-Resolved Photoluminescence. *Physical Review Applied* **6**, 1–13 (2016).
33. Stolterfoht, M. *et al.* How To Quantify the Efficiency Potential of Neat Perovskite Films: Perovskite Semiconductors with an Implied Efficiency Exceeding 28%. *Advanced Materials* **32**, 2000080 (2020).
