# Supplementary material for: Open-circuit and short-circuit loss management in wide-gap perovskite p-i-n solar cells
Source: Nat Commun. 2023 Feb 20;14:932. doi: 10.1038/s41467-023-36141-8 (PMC9941504; doi:10.1038/s41467-023-36141-8)
Supplement: Supplementary file 3 — Solar Cells Reporting Summary [file 41467_2023_36141_MOESM3_ESM.pdf]

nature research

Corresponding author(s): Henry Snaith, Pietro Caprioglio

Solar Cells Reporting Summary

Nature Research wishes to improve the reproducibility of the work that we publish. This form is intended for publication with all accepted papers reporting the characterization of photovoltaic devices and provides structure for consistency and transparency in reporting. Some list items might not apply to an individual manuscript, but all fields must be completed for clarity.

For further information on Nature Research policies, including our data availability policy, see Authors & Referees.

Experimental design

Please check: are the following details reported in the manuscript?

1. Dimensions

Item	Yes/No	Details
Area of the tested solar cells	☒ Yes ☐ No	The full dark areas of the cells are 0.309 cm2 and 1.139 cm2. The masked areas of the cells are 0.25 cm2 and 1 cm2.
Method used to determine the device area	☒ Yes ☐ No	Optical microscope

2. Current-voltage characterization

Item	Yes/No	Details
Current density-voltage (J-V) plots in both forward and backward direction	☒ Yes ☐ No	Scan and reverse scans are reported.
Voltage scan conditions<br>*For instance: scan direction, speed, dwell times*	☒ Yes ☐ No	The used scan speed is approximately 30 mV/s in both forward and reverse direction. Steady-state maximum power point is also reported for each device condition.
Test environment<br>*For instance: characterization temperature, in air or in glove box*	☒ Yes ☐ No	Cells are measured in air environment at room temperature condition.
Protocol for preconditioning of the device before its characterization	☐ Yes ☒ No	We did not use any preconditiong protocol.
Stability of the J-V characteristic<br>*Verified with time evolution of the maximum power point or with the photocurrent at maximum power point; see ref. 7 for details.*	☒ Yes ☐ No	Steady-state maximum power point tracking is reported for each device condition in SI. For the champion device steady-state maximum power point tracking is reported also in the main text.

3. Hysteresis or any other unusual behaviour

Item	Yes/No	Details
Description of the unusual behaviour observed during the characterization	☒ Yes ☐ No	Exemplary hysteresis are reported for each device condition.
Related experimental data	☒ Yes ☐ No	Typical hysteresis of devices is reported both in the main text and in SI.

4. Efficiency

Item	Yes/No	Details
External quantum efficiency (EQE) or incident photons to current efficiency (IPCE)	☒ Yes ☐ No	The EQE data are reported in SI.
A comparison between the integrated response under the standard reference spectrum and the response measure under the simulator	☒ Yes ☐ No	The integrated short-circuit current from the EQE spectrum matches the short—circuit current from the JV scan (of masked cells) within 1%.
For tandem solar cells, the bias illumination and bias voltage used for each subcell	☐ Yes ☒ No	We did not fabricate any tandem device.

5. Calibration

Item	Yes/No	Details
Light source and reference cell or sensor used for the characterization	☒ Yes ☐ No	AM1.5G irradiance generated by a Wavelabs SINUS-220 solar simulator calibrated with a certified KG3-filtered reference diode (Fraunhofer). The spectral mismatch factor was estimated to be 0.006.
Confirmation that the reference cell was calibrated and certified	☒ Yes ☐ No	The solar simulator was calibrated with a KG3-filtered reference diode (Fraunhofer)

November 2017

Calculation of spectral mismatch between the reference cell and the devices under test

- [x] Yes
- [ ] No

A spectral mismatch calculation was based on the spectral irradiance of the solar simulator, the EQE of the reference silicon solar cell and typical EQE of our cells. This resulted in a mismatch factor of 0.006.

6. Mask/aperture

Size of the mask/aperture used during testing

- [x] Yes
- [ ] No

Mask size 0.25 cm2 and 1 cm2

Variation of the measured short-circuit current density with the mask/aperture area

- [x] Yes
- [ ] No

All short-circuit currents were measured always on masked devices.

7. Performance certification

Identity of the independent certification laboratory that confirmed the photovoltaic performance

- [ ] Yes
- [x] No

No external certifican was performed given that no record efficiencies for perovskite solar cells were reached.

A copy of any certificate(s)
Provide in Supplementary Information

- [ ] Yes
- [x] No

No external certifican was performed given that no record efficiencies for perovskite solar cells were reached.

8. Statistics

Number of solar cells tested

- [x] Yes
- [ ] No

Overall we fabricated a total of roughly 300 devices. We reported the full statistic with a PCE cut off applied for devices with PCE lower than half of the mean value.

Statistical analysis of the device performance

- [x] Yes
- [ ] No

Statistical analysis is reported for each device condition.

9. Long-term stability analysis

Type of analysis, bias conditions and environmental conditions
For instance: illumination type, temperature, atmosphere humidity, encapsulation method, preconditioning temperature

- [x] Yes
- [ ] No

Long-term stability measurements (>3500 h) were performed under open-circuit for non-encapsulated devices under N2 atmosphere at 85 C.
